# **Adhesion Reinforcement of Electrode**–**Electrolyte Interface in Flexible Electrochemical Energy Storage Devices**

**DOI:** 10.1007/s40820-026-02084-0

**Published:** 2026-02-17

**Authors:** Xian Xie, Qiuhong Wang, Faheem Mushtaq, Kelong Ao, Hong Zhao, Walid A. Daoud

**Affiliations:** 1 Department of Mechanical Engineering, City University of Hong Kong, Hong Kong, People’s Republic of China; 2 Centre for Cooperative Research on Alternative Energies, Basque Research and Technology Alliance, Vitoria-Gasteiz, Spain

**Keywords:** Flexible batteries, Wearable electronics, Adhesion, Interface, Deformability

## Abstract

Wearable and deformable electronics are becoming increasingly essential components of modern healthcare and daily life. To power such devices, flexible electrochemical energy storage (FEES) plays a critical role. The practical performance of FEES is dominated by charge and mass transfer at the electrode-electrolyte interface, similar to many rigid battery technologies. However, a unique challenge for FEES is the durability of this interface under deformation. Herein, we present the first comprehensive review of the interface physics, unveiling the crucial role of interface adhesion in the mechanical endurance of FEES. By bridging adhesion physics, material chemistry, and device mechanics, adhesion reinforcement strategies are comprehensively discussed and quantitatively compared, providing multi-scale mechanisms for optimizing FFES interface - from nanoscale bond engineering to microscale surface topology, mechanical interlocking, and macroscale device design. Further, inspired by the synergetic effect of adhesion mechanisms, we propose potential research directions for durable electrode-electrolyte interfaces under dynamic deformation. We also revisit the evaluation of flexibility and electrochemical performance, proposing an application-driven bending index for device assessment. These insights on electrode-electrolyte interface physics of FEES will facilitate the flourishing future of flexible devices.

## Highlights

- This article is the first to systematically bridge adhesion physics, materials science, and device mechanics in flexible electrochemical energy storage, offering a multi-scale perspective from nanoscale bonds to macroscale design.
- Comprehensive adhesion reinforcement strategies are classified and compared, with explicit emphasis on interfacial durability under dynamic deformation.
- A novel application-driven bending index is introduced for standardizing the evaluation of flexible electrochemical energy storage flexibility, providing a practical framework for device assessment and comparison.

## Graphical abstract

**Figure:**
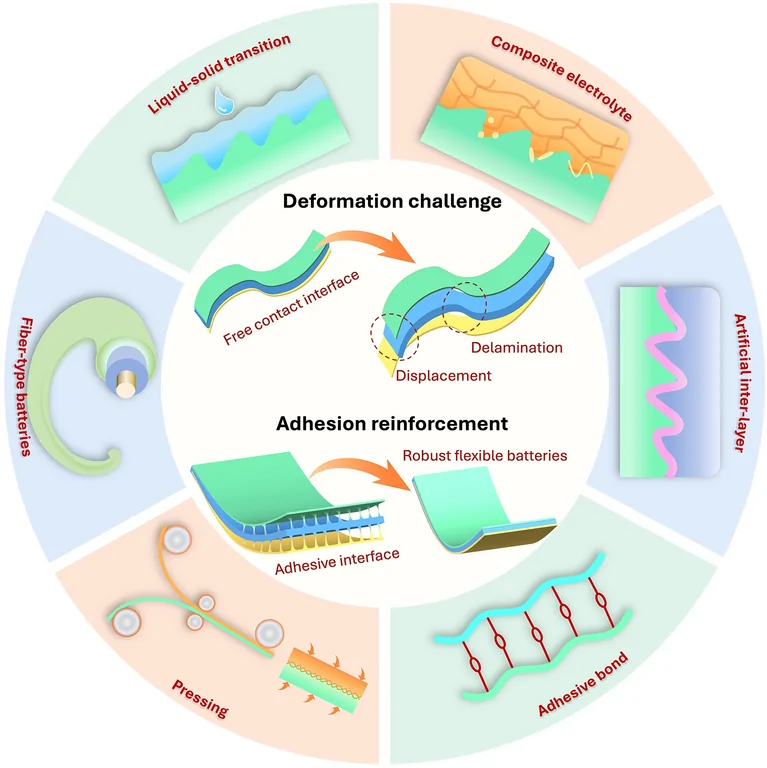

## Introduction

**Fig. 1:**
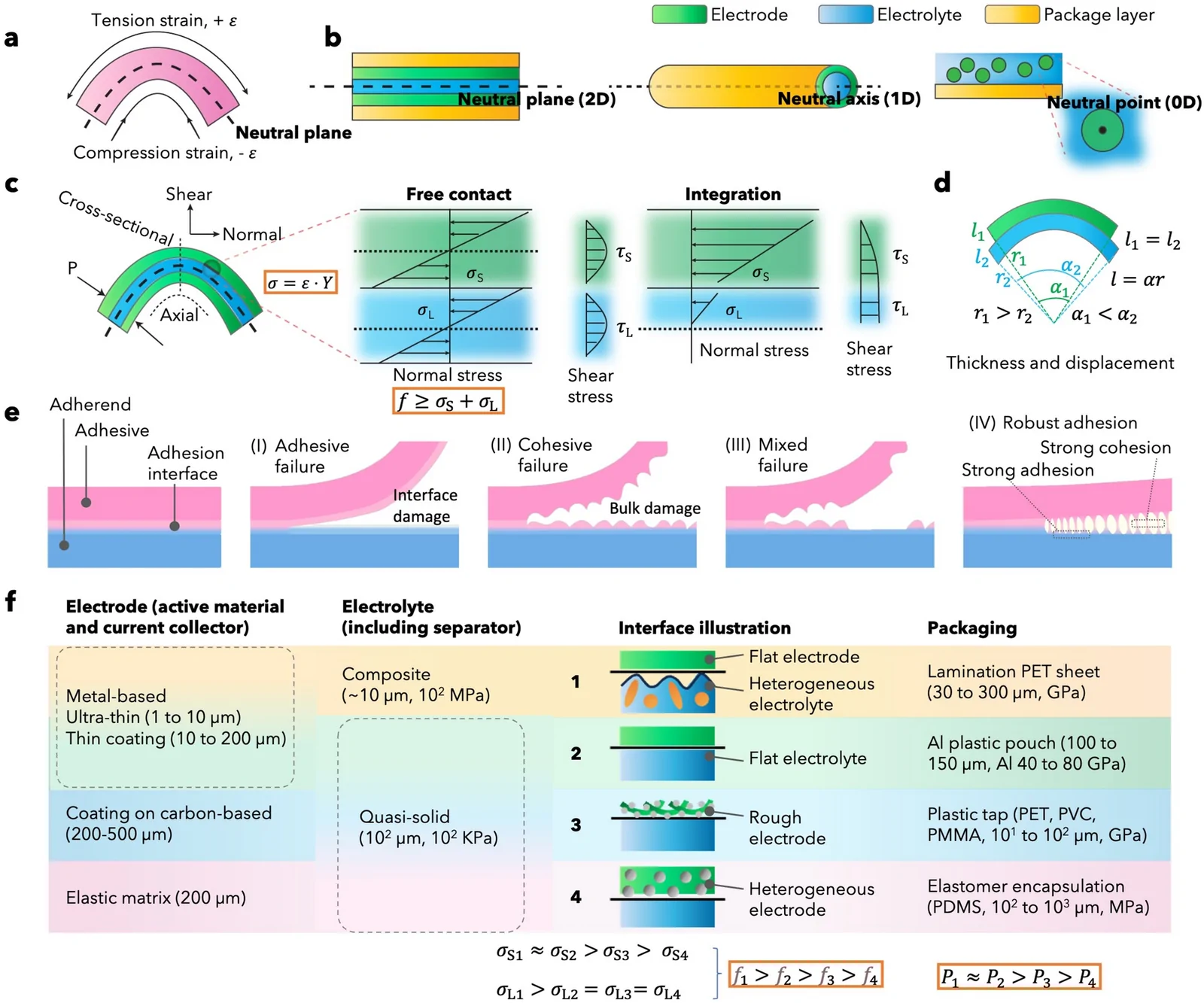
Mechanical analysis of FEES and corresponding classification.** a** Neutral plane at bending state. **b** Neutral plane design principal application in planar, fiber-type and intrinsic stretchable FEES. **c** Strain stress illustration at bending state. *P*: perpendicular pressure provided by packaging; *σ*: normal stress in the cross-section of the beam induced by the axial strain *ε*; *σ*_S_: the stresses in the electrode; *σ*_L_: the stresses in the electrolyte; *Y*: Young’s modulus; *f*: adhesion strength). **d** Thickness impact on displacement. *l*: device length; *r*: radius; *α*: sector angle. When wrapping the device in a cylindrical surface, the larger layer thickness induces larger difference in *r*_1_ and *r*_2_*,* resulting in relative displacement of layers. **e** A basic adhesion model involves an adherend, adhesive, and simultaneously generated adhesive interface of adhesive joints. **f** Mechanical properties of electrode, electrolyte and packaging materials of FEES lead to four mechanic models with different requirements of electrode–electrolyte interfacial adhesion

From deformable displays to wearable sensors, modern consumer electronics are becoming increasingly flexible, and not just in terms of the features offered. To achieve fully flexible devices, all components must be flexible, including the power supply and energy storage. Thus, there is a booming need for flexible electrochemical energy storage (FEES). The electrochemistry of FEES is similar to that of conventional rigid batteries. However, the dynamic operating conditions create unique challenges. During bending, a FEES device experiences tensile strain on the convex surface and compression on the concave surface, with a central neutral plane without strain (Fig. 1a). Positioning brittle components in this isotropic zone minimizes strain-induced degradation.

Three main FEES configurations have arisen (Fig. 1b). Planar FEES is a layer-by-layer configuration with electrodes located in the two-dimensional neutral plane, encapsulated by flexible layers (electrolyte and/or packaging layers) [1, 2]. Fiber-type FEES, which adopts a one-dimensional neutral axis design, is compatible with textile engineering. The zero-dimensional neutral-point design can achieve intrinsic stretchable FEES, which involves downsizing the bending mechanics to the microscale, where particles of the active electrode material can be dispersed in and encapsulated by the elastomer matrix [3]. Complex geometries that offer structural flexibility, such as wavy or folded origami designs that locate strain on the joints, can be analyzed at the local level using a combination of the above three categories [4].

In all three FEES configurations, it is essential to optimize the interlayer adhesion. Unlike brittle electrodes, FEES electrolytes are flexible or even stretchable and can therefore survive the strain generated outside the neutral zone. However, different strains in the electrode and electrolyte cause interfacial displacement if adhesion is suboptimal, diminishing the close contact and electrochemical active area. Adhesion also facilitates stress transfer between neighboring layers, protecting the electrodes and enhancing the flexibility of devices [5]. Despite its importance, adhesion in FEES is often overlooked in the literature, because the multilayer configuration is usually assumed to be well-integrated. Therefore, we examine the mechanics of multilayer FEES under bending, illustrating adhesion requirements to prevent displacement. This work aims to analyze the current literature on adhesion combined with adhesion science, focusing on three aspects that have not been addressed in previous works: (1) Comprehensively reviewing adhesion reinforcement strategies for planar and fiber-type FEES, and discussing their role and limitations. Early efforts to improve adhesion relied on qualitative methods, and it was not until 2023 that studies provided electrochemical indicators correlated with quantitative adhesion. Hence, we provide a timely table that summarizes research achievements of interface adhesion for FEES. In addition, interfacial physics and chemistry can be modified synchronously when adhesion reinforcement is applied, which is also thoroughly considered and discussed. (2) Elucidating adhesion mechanisms of the strategies from macroscale to nanoscale. (3) Introducing a new evaluation index for the flexibility and durability of FEES and a multicriteria assessment framework. Finally, we conclude the opportunities for future research.

## Interface Adhesion Requirement of FEES

To illustrate the adhesion requirement against interfacial displacement under deformation, the stress distribution on the electrode–electrolyte interface of a multilayer configuration is analyzed as shown in Fig. 1c. The axial strain, *ε*, induces normal stress, *σ*, in the cross-section of the beam. The stresses in the electrode, *σ*_S_, and electrolyte, *σ*_L_, are different due to their distinct Young’s modulus, *Y*. Assuming free contact between electrode and electrolyte, the neutral planes exist in each layer and the sliding force between two layers is the sum of *σ*_S_ and *σ*_L_. Layer displacement is caused by the sliding force which is significantly affected by thickness (Fig. 1d), and the radius of the outer layer (${r}_{1}$) is larger than that of the inner layer (${r}_{2}$) due to the layer thickness simultaneously resulting in different central angles (${\alpha }_{1}$ and ${\alpha }_{2}$) representing the different bending status. For ultra-thin layers (${r}_{1}$ is very close to ${r}_{1}$), the bending status of the two layers is close leading to a small displacement. Thus, reducing the thickness of components is an effective strategy to increase flexibility. However, achieving practical areal capacity in FEES requires high mass loading, which inevitably leads to thicker electrodes and associated mechanical penalties, such as higher bending strain and stress concentration [6]. In addition, the different mechanical properties lead to inhomogeneous strain and stress distribution across layers further exacerbating delamination. As a result, FEES face a unique design trade-off between high areal capacity and mechanical flexibility, which underscores the critical role of interface adhesion engineering, and stress-dissipating architectures, such as natural plane and geometry design, to enable mechanically durable performance at practical mass loadings. Therefore, the adhesion strength, *f*, needs to overcome the sliding force and sufficient *f* will integrate the electrode and electrolyte forming one neutral plane, where the interior stress can transfer from the neutral plane to outer layers, modulating the stress distribution. In multilayer devices, the neutral plane is determined by Young’s modulus and the thickness of each layer. With appropriate design and adhesion, fragile and stiff components, generally electrodes, can be placed in the no-strain neutral plane, while soft and elastic encapsulation with suitable thickness bears stress, achieving ultra-high flexibility. Of note, the elastic modulus of each layer should be considered for compliance [3]. Adhesion offsets the mismatch modulus of components to some extent, where a larger mismatch requires higher adhesion [7, 8]. Although strong and continuous interfacial adhesion enables efficient stress transfer toward the packaging layer, whose higher modulus and plastic deformability should be able to dissipate external loads, this assumes that the active electrode can sustain the transmitted stresses without structural failure. In practice, many cathode materials are inherently brittle and have low fracture strength (< 1 MPa) [9]. This brittleness is exacerbated in high-energy–density cells, where thicker and higher-mass-loading electrodes further increase bending-induced strain and stress concentration [5]. When the interfacial adhesion is excessively strong, the electrode is fully coupled to the deformation of the cell and may experience stress levels that exceed its fracture threshold, leading to severe cracking. In contrast, an interface with optimized adhesion and internal cohesion allows reversible microsliding or microcracking, which provides local strain relief while preserving electrical contact and electrochemical performance. Therefore, the adhesion level must be carefully balanced to enable stress transfer while accommodating the intrinsic mechanical limitations of brittle electrode materials.

Adhesion is an intermolecular force between two different substances, while cohesion occurs within a single substance (Fig. 1e). Failure analysis identifies weaknesses in bulk material or adhesive joints. Cohesion depends on mechanical and viscoelastic properties, while adhesion relates to energetic parameters of the adhesive and adherend [10]. Adhesive failure occurs when cohesive strength exceeds interfacial strength. Cohesive failure occurs when interfacial strength exceeds cohesive strength. In most cases where interfacial strength is close to cohesive strength, adhesive failure is accompanied by cohesive failure (mixed failure). A balance between cohesion and interfacial adhesion is critical for durability [11]. Ideal adhesion requires (1) a clean surface without a weak boundary layer; (2) good wetting influenced by surface energy, morphology, and liquid surface tension; (3) solidification through chemical reaction, phase changes, or solvent evaporation. Adhesion assessments include tensile test for layer cohesion, lap-shear test for adhesive strength, and peel test for interface toughness and resistance to delamination. In addition to these static adhesion tests, adhesion can progressively degrade under loading fatigue, where cyclic deformation induces gradual loss of interfacial contact, microvoid growth, bond fatigue, and breakdown of mechanical interlocking. Therefore, fatigue-cycling tests should also be implemented to evaluate the long-term durability and reliability of adhesion. Environmental temperature and moisture also influence adhesion. Elevated temperature can soften polymeric adhesives (lowering their modulus and increasing chain mobility), which reduces their load-bearing capability leading to interfacial slippage or debonding. While low temperatures can stiffen polymers, making the interface more brittle and prone to crack initiation under bending. Moisture degrades adhesion by altering surface energy and/or volume expansion diluting adhesive bonding/interactions and disrupting mechanical interlocking.

Multilayer FEES are classified into four mechanical models based on electrode and electrolyte materials and interfacial adhesion demands (Fig. 1f). Models 1 and 3 describe interfaces formed between a flat surface and a rough surface, but differ in which layer provides the roughness. In Model 1, a flat electrode contacts a heterogeneous composite electrolyte containing rigid solid compounds. The solid–solid microstructure of the composite electrolyte produces a relatively rough and mechanically stiff surface. In contrast, Model 3 involves a rough and porous carbon-based electrode in contact with a soft and homogeneous quasi-solid electrolyte whose liquid-rich phase gives it an even and compliant surface. For both models, surface roughness and wetting property govern interfacial adhesion. When the contact angle < 90°, surface roughness improves wetting, while the opposite effect occurs when it > 90° [12]. Surface roughness increases the contact area, enhancing intermolecular attraction and preventing crack propagation [10]. For good adhesion on rough surfaces: 1) If the adhesive substance is liquid/gel-like, the liquid should have low-surface tension and suitable fluidity to fill voids and 2) if the adhesive substance is solid/semi-solid, the modulus of the adhered and adhesive should allow mechanically interlocking ensuring intimate contact. Model 2 describes the interface formed between two flat-even surfaces, typically a flat electrode and a homogeneous quasi-solid electrolyte. Because mechanical interlocking is minimal in this model, adhesion depends primarily on interfacial energy matching, in which surface tension, polarity, and specific chemical interactions (e.g., ion–dipole or hydrogen bonding) determine whether adhesion is uniform across the flat substrate. Model 4 represents the adhesion between a flat electrolyte and an electrode with a neutral-point design, where cohesion is vital to maintain discontinuous electrode particles embedded in an elastomer matrix. A mismatch between cohesion and adhesion can result in electrode particles detachment or electrolyte damage leading to breakage of the interface. Thus, Model 4 demands a balanced adhesion and cohesion to maintain mechanical integrity. The interface of fiber-type FEES is rarely discussed in previous literature, which will also be interpreted in this review. In general, the interface adhesion strength of Model 1 to 4 should follow the order *f*_1_ > *f*_2_ > *f*_3_ > *f*_4_. The perpendicular pressure from packaging layers (*P*) physically forces layers connected compensating for the interface adhesion, which varies for different materials, thickness, and fabrications.

## Interface Reinforcement Strategies

**Fig. 2:**
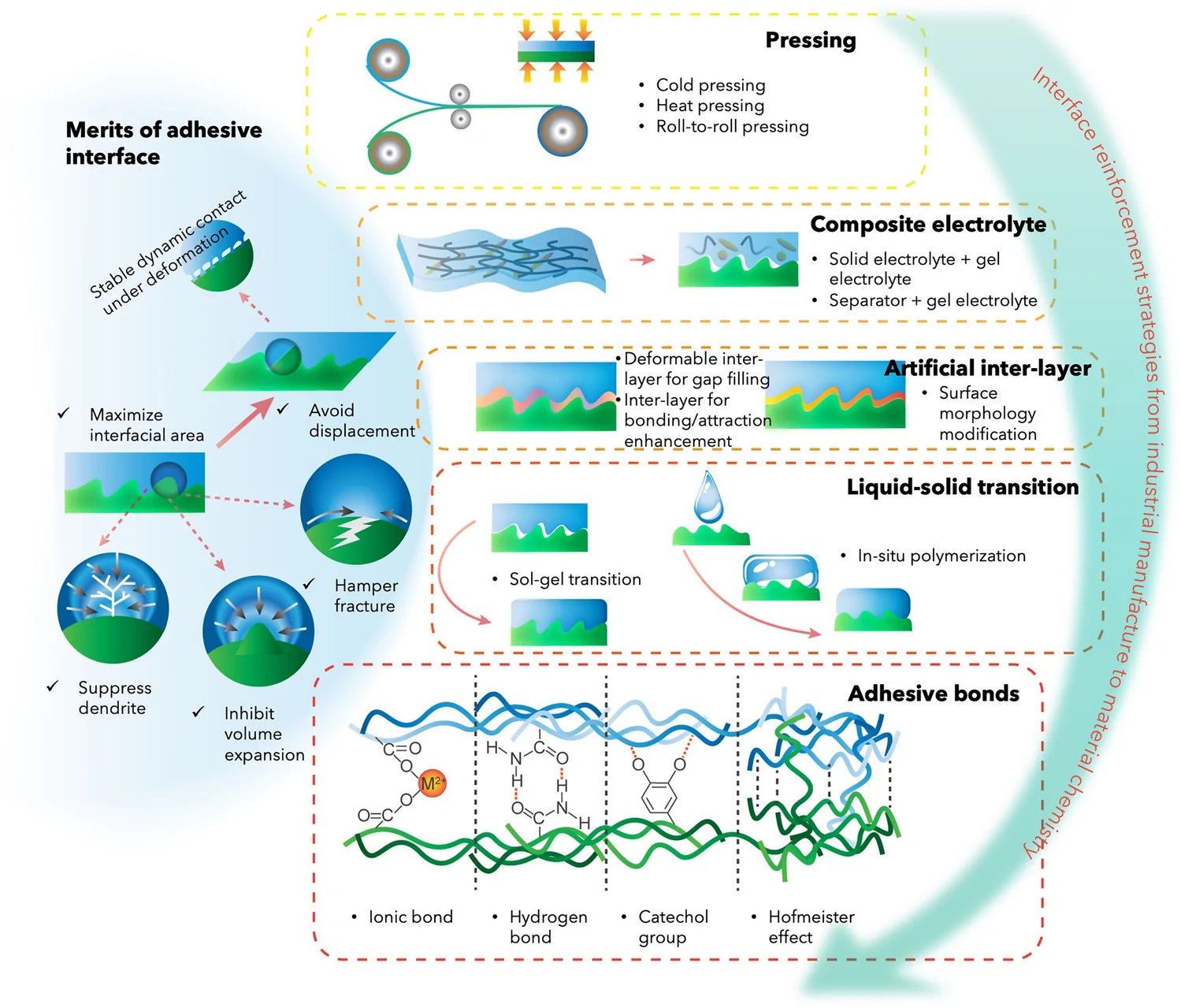
Merits of adhesive interface and reinforcement strategies for planar FEES. The reinforcement strategies are classified into five groups from component scale to nanoscale

Interface adhesion between components allows efficient and uniform ion transport. Strong adhesion at the electrolyte–anode interface suppresses dendrites through mechanical pressure, whereas at the cathode–electrolyte interface, it prevents volume expansion and fractures. The key ways to reinforce adhesion are enlarging the contact area (creating a more adhesive joint) and enhancing adhesion intensity. We categorized reinforcement strategies for planar FEES into five groups: pressing, composite electrolyte, artificial interlayer, liquid–solid transition, and adhesive bonds (Fig. 2). For fiber-type FFES, the interface challenge and fabrication are distinct from the planar FFES. Thus, the reinforcement strategies for fiber-type FEES are separately discussed.

### Reinforcement Strategies for Planar FEES

#### Pressing

**Fig. 3:**
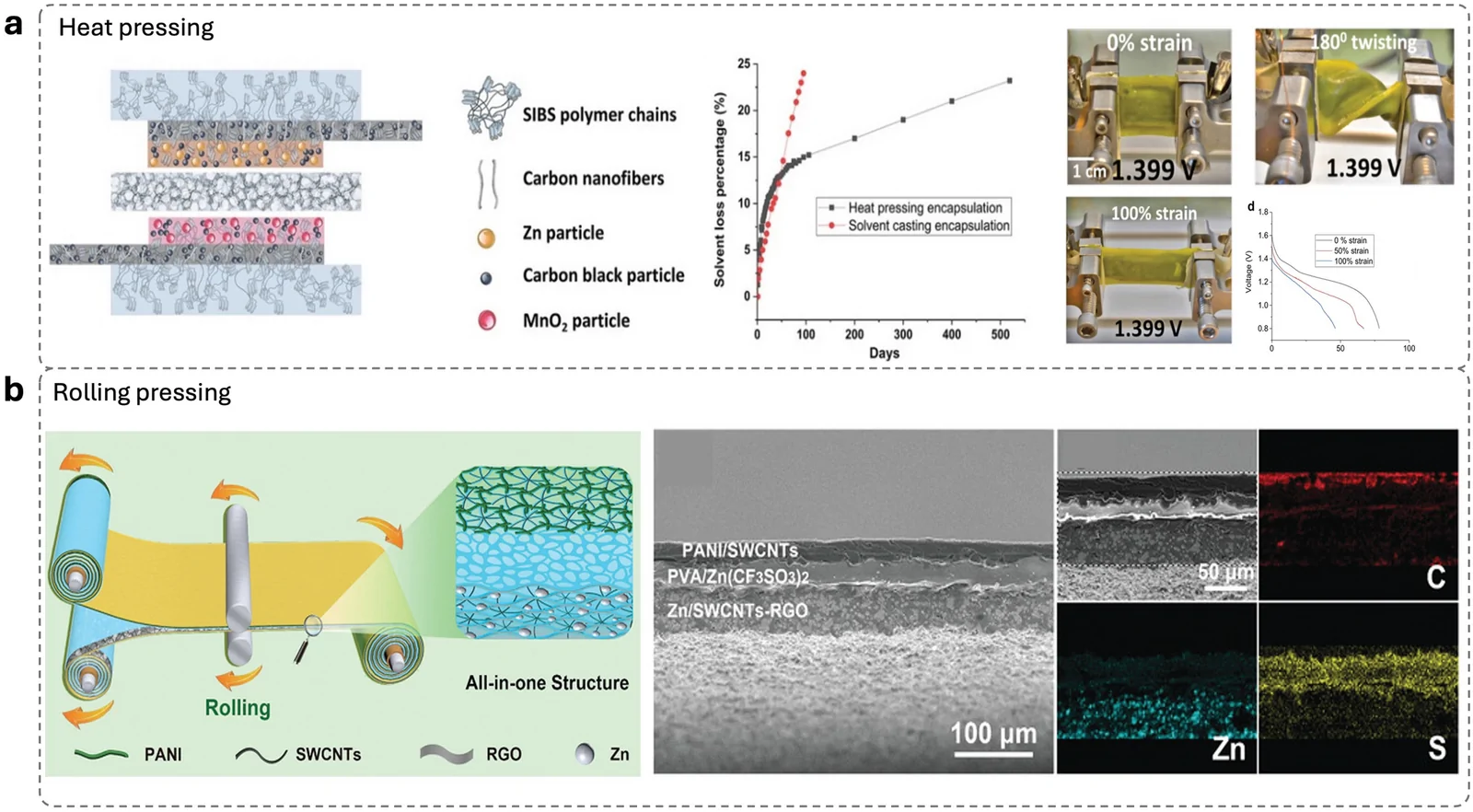
Integrated devices using pressing approach. **a** Heat-pressing for an integrated Zn-ion battery with styrene-based polymer framework [15]. Reproduced with permission. Copyright 2021, The Author(s) and Wiley‐VCH GmbH. **b** Scalable rolling pressing and the resulted intimate interface [16]. Reproduced with permission. Copyright 2021, Wiley‐VCH GmbH

Pressing is a straightforward way to reinforce the physical contact between an electrode and an electrolyte, which is adaptive to Models 1, 2 (with a thin and robust electrolyte), and 4. The interface wettability and polymer entanglement can be enhanced by high-pressure pressing. The rolling press is suitable for flexible device fabrication and is highly scalable, while plate compressing is more practical for lab-scale studies, and its application is limited to separator-assisted FEES. Roll-to-roll pressing has been employed to integrate the anode and cathode, which were pre-spun fibrous gel polymer electrolytes. The demonstrated flexible Li-ion battery exhibited stable open-circuit voltage at a fully charged state after bending cycles [13]. Although the electrode–electrolyte interface was initially formed when spinning the electrolyte on the electrode, the subsequent pressing strengthened the interface contact and adhesion. It is noteworthy that the pre-spun electrolytes on both electrodes contain the same polymer, benefiting the generation of interfacial chemical bonds and polymer entanglement during the pressing. Likewise, the common network in the electrode and electrolyte has also been utilized in a flexible supercapacitor assembled by pressing [14]. A styrene-based polymer has been used as the common framework for multiple layers in a flexible Zn-ion battery, resulting in an integrated configuration by heat-pressing, presenting stable discharge capacity after 150 stretching cycles at 100% strain (Fig. 3a) [15]. The tight integration granted by heat-pressing largely reduces electrolyte evaporation and allows the cell to withstand washing in a home washing machine (Fig. 3a). Moreover, a scalable rolling press approach has been developed using PVA-based gel electrolytes demonstrating a continuous and seamless interfacial connection (Fig. 3b) [16]. However, roll-to-roll compressing is not suitable for materials with low mechanical strength, such as hydrogel electrolytes. Thus, we propose vacuum sealing as the alternative method for fragile materials to provide shape-adaptive and continuous compression. The adhesion mechanism of pressing strategies is polymer entanglement or topological adhesion. In particularly, mechanical interlock and adhesive bonds can be formed during heat-pressing and aging processes, respectively.

#### Composite Electrolyte

**Fig. 4:**
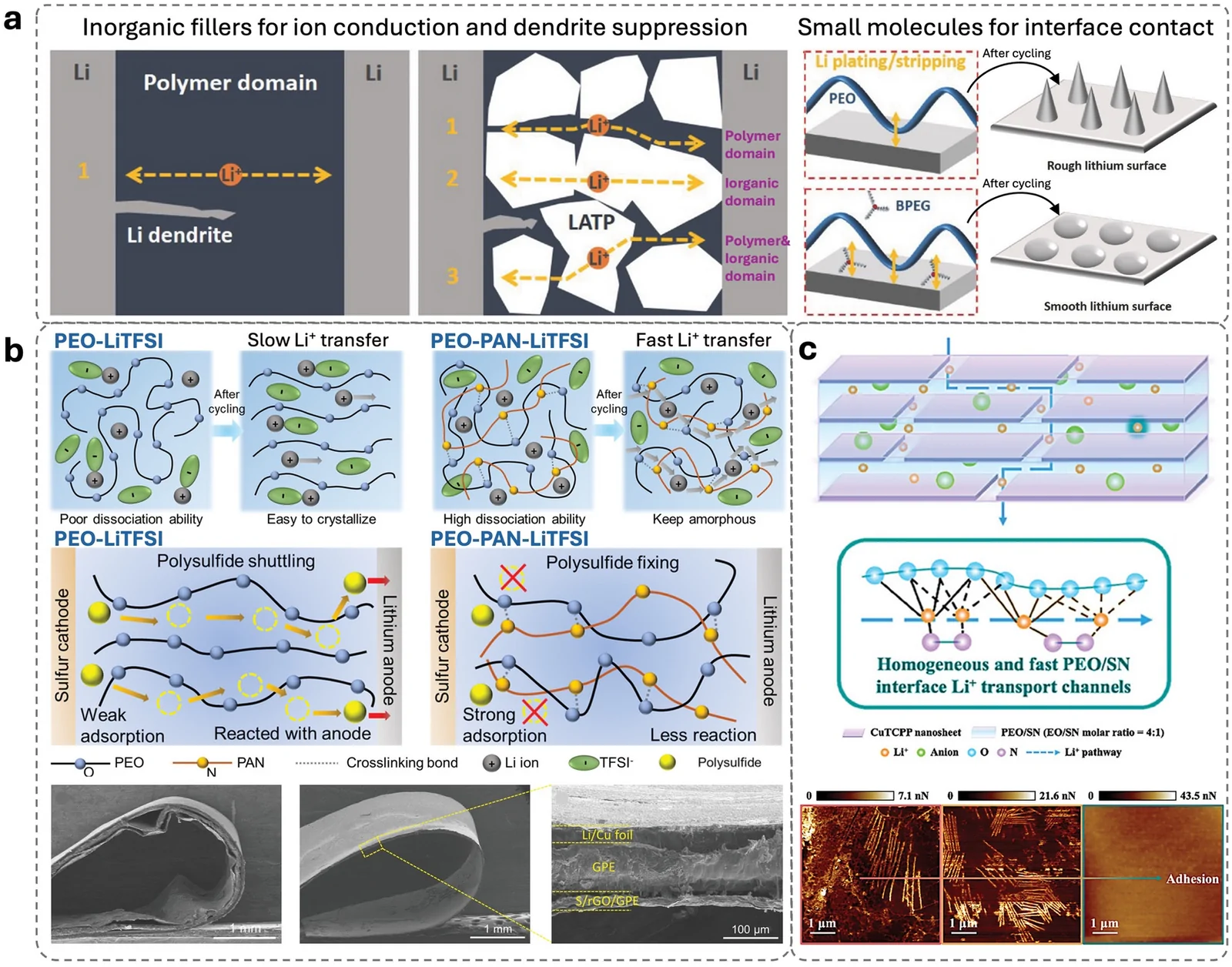
Multifunctional composite electrolytes.** a** Functions and improvement mechanisms of inorganic fillers and small molecule additives, respectively [17]. Reproduced with permission. Copyright 2017 WILEY‐VCH Verlag GmbH & Co. KGaA, Weinheim. **b** Copolymer network of PAN fibers crosslinked PEO matrix for fast transferring Li^+^ while fixing polysulfide and tight contact [22]. Reproduced with permission. Copyright 2022 Wiley‐VCH GmbH. **c** Viscous-fluid ionic conductor filled into laminar framework and the resultant adhesive interface [24]. Reproduced with permission. Copyright 2024 The Author(s) and Wiley‐VCH GmbH

Composite electrolytes integrating inorganic fillers in a polymer matrix have been widely used in Model 1 for non-aqueous solid-state FEES, where the ionic conductivity and chemical stability are improved by the inorganic components, and the polymer provides mechanical flexibility and a soft and intimate interface. To enhance the interfacial contact at the initial state, smaller molecules or wetting agents can be introduced to fill the voids between the composite electrolyte and the electrode [17]. A composite electrolyte consisting of polymer chains, inorganic solid particles and oligomers was developed for a Li-ion battery, where the function of the inorganic filler and the oligomer additive was proposed. As shown in Fig. 4a, the compact inorganic filler provides continuous ion conduction and a tough physical barrier to mechanically prevent the growth of dendrites. The oligomer additive with a relatively small molecule size fills into the voids between the polymer chain and the electrode improving the interface contact. To enhance interface stability during cycling, various microscale three-dimensional (3D) networks have been investigated to suppress dendrite and side reactions at the anode side and buffer volume changes at the cathode side [18–20]. These microscale networks formed by nanowires or nanofibers can improve adhesion by mechanical interlock [21]. For example, a flexible Li-ion battery using electrolyte of Li_0.35_La_0.55_TiO_3_ (LLTO) nanowires-filled PVDF with LiClO_4_ was demonstrated displaying satisfactory cycling capacity at static bending and folding states, in which cellular structure presented great ability to withstand stress and buffer the mechanical expansion leading to good interfacial contact during repeated deformation [20]. Moreover, polyacrylonitrile nanofibers (PAN) were introduced into poly(ethylene oxide) (PEO) matrix forming copolymer membrane (Fig. 4b), where the interconnected copolymer structure reduced the crystallinity of the membrane accelerating the Li^+^ ions transfer, and the strong C = N–O bonding between the copolymer and electrodes suppress the Li_2_S_6_ molecules shuttling at the cathode, granting the Li–S battery 96.4% capacity retention after 1000 bending cycles [22]. Further, the high bonding strength of the copolymer membrane enables the cell to have a crack-free and tight contact under bending. A composite electrolyte with high NASICON content (80 wt%) was obtained with an ionic liquid wetting agent, resulting in improved room-temperature ionic conductivity (1.48 × 10^–4^ S cm^−1^) [23]. Although high-solid content improves ionic conductivity and mechanical strength for fast ion transport and dendrite inhibition, poor solid–solid contact is concerning for the interface construction in high-solid content composite electrolyte. To balance the trade-off between mechanical modulus and adhesion, viscous-fluid ionic conductor was introduced to laminar framework forming a composite electrolyte for a Li-metal battery (Fig. 4c), and the adhesiveness of the viscous-fluid ionic conductor constructed an adhesive contact between the composite electrolyte and the electrodes which enabled the interface a fast Li-ion transfer kinetic and sufficient mechanical strength for dendrite suppression simultaneously [24]. Atomic force microscopy was used to assess the adhesion force providing direct and quantitative characterization of the interface adhesion [24]. Low room-temperature ionic conductivity remains the bottleneck of solid-state FEES, due to the sluggish ion movement in the bulk and/or poor interfacial contact.

In general, composite electrolytes with high-solid content have high stiffness and must be made thin to be suitable for flexible applications. However, thin membranes have limited stretchability, making them unsuitable for wearable planar FEES. Alternatively, the membrane can be wrapped around electrode fibers in a fiber-type FEES. Nevertheless, the multifunctionality of composite electrolytes provides valuable insights into factors such as dendrite suppression and cathode immobilization, which then can be transferred to future FEES studies to realize a versatile interface [21, 25].

#### Artificial Interlayer

**Fig. 5:**
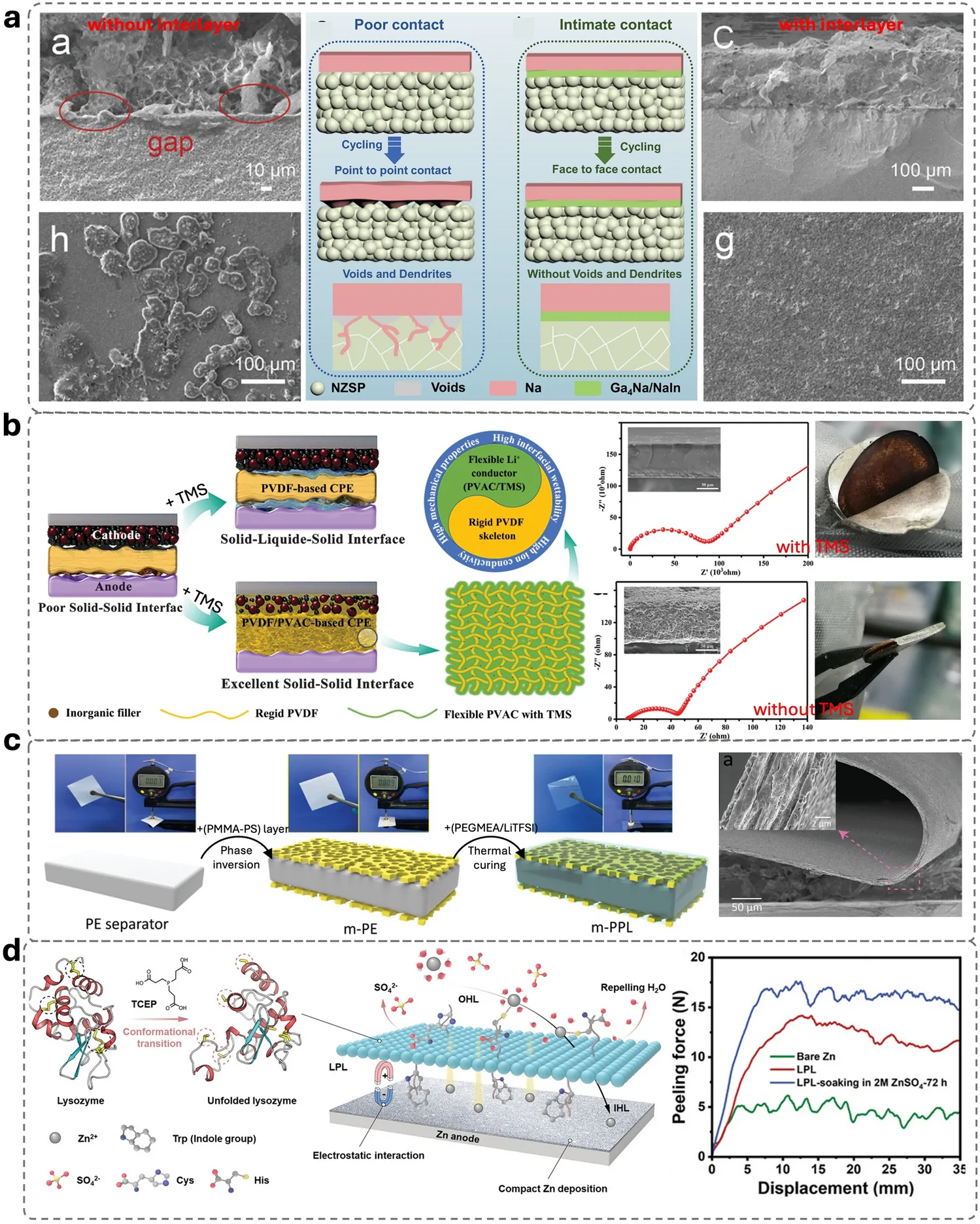
Artificial interlayer for interface wettability and compatibility.** a** Liquid alloy interlayer for intimate interface contact and resultant top surface and cross-sectional morphology after repeated Na plating/stripping [30]. Reproduced with permission. Copyright 2024 Wiley‐VCH GmbH. **b** Rigid-flexible coupling electrolyte employed a TMS selective wetting agent, and the resultant impedance decrease and tight connection after Li plating/stripping [32]. Reproduced with permission. Copyright 2020 WILEY‐VCH Verlag GmbH & Co. KGaA, Weinheim. **c** Gel polymer interlayer for improving the interface compatibility and connection between electrolytes and electrodes [28]. Reproduced with permission. Copyright 2021 Wiley‐VCH GmbH. **d** Adhesive lysozyme protection layer on Zn anode for dendrite and HER [37]. Reproduced with permission. Copyright 2024 Wiley‐VCH GmbH

An artificial interlayer can achieve a compatible interface within a specific region without compromising the flexibility of the device, which has been applied to Models 1 and 3. Gel polymer is a common choice for the interlayer, serving different objectives, such as decreasing interfacial resistance [26], mitigating volume variations of cathode materials [27], ensuring solid–electrolyte interface compatibility [28], and regulating ion transport for uniform metal deposition [29]. Good wetting is essential for forming plentiful adhesive joints, which largely depends on surface tension (for liquids) or surface energy (for solids). A wettable liquid alloy interlayer was implemented between Na metal anode and Na_3_Zr_2_Si_2_PO_12_ cathode to address the poor interfacial contact issue in a solid-state Na-battery (Fig. 5a), where the excellent wettability and strengthened adhesion reduced void and dendrite formation [30]. The wettability resulted from a thin Ga_2_O_3_ layer. The adhesion is induced by the spontaneous chemical reaction of GaIn with Na metal and the low interfacial formation energies of the cathode/Ga_4_Na and Na/Ga_4_Na confirm it as a good adhesive between the cathode and the Na anode. A similar effect was observed for the Li_x_Sn hybrid interlayer [31]. Rigid-flexible coupling electrolyte is a popular design for solid-state lithium-based batteries because it provides both sufficient Li^+^ conductivity for low resistance and mechanical properties for dendrite inhibition. By sophisticated matching, a wetting agent can be found to selectively combine with the flexible counterpart in a composite electrolyte without affecting the high mechanical strength of the rigid counterpart. Tetramethylene sulfone (TMS) can selectively wet polyvinyl acetate (PVAC) in a PVDF-PVAC composite electrolyte (Fig. 5b), forming a soft solid–electrolyte interface with high Li^+^ conductivity to solve the poor interfacial compatibility between PVDF-based electrolyte and electrodes while maintaining the rigidity of the PVDF skeleton [32]. Following a similar concept, the ceramic electrolyte and electrodes (Li anode and LiFePO_4_ cathode) can be bridged by a sticky PEO interlayer. The PEO perfectly fills the gap between the ceramic electrolyte and the solid electrode owing to its high flexibility and affinity. The design of the flexible interlayer can be customized according to the requirements of the cathode–electrolyte interface and the anode–electrolyte interface to achieve distinct functions [29, 33, 34]. It is worth noting that the artificial interlayer can be established on the electrolyte or electrode [26]. Fluoroethylene carbonate modification is applied on Li anode to protect the organic content in the electrolyte from the reaction with Li and form a stable solid–electrolyte interphase (SEI) layer, which results in a high interfacial compatibility and stability [34–36]. High-capacity retention after 120 bending cycles was reached by a flexible Li-ion battery using both gel-covered anode and cathode [27]. A compact layer configuration without delamination under a static bending state was demonstrated in a flexible Li-metal battery with gel polymer interlayer between a modified polyethylene separator and electrodes (Fig. 5c) [28]. Such an interlayer favors adhesion by chemical bonding between the polymer and the electrode, or even mechanical interlocking when in situ polymerized in a porous configuration.

To date, artificial interlayer has mainly been applied in non-aqueous FEES, the polymer electrolyte of which has intrinsically low conductivity, poor interfacial adhesion, and low compatibility with electrode materials. For aqueous FEES, water is a good medium for fast ion transport and an excellent solvent, in which the modifications are commonly applied to the bulk forming homogeneous hydrogel electrolytes. However, recent studies of aqueous zinc-based FEES have highlighted a low negative/positive (N/P) ratio, which requires eliminating side reactions induced by active water molecules, such as zinc corrosion and hydrogen evolution. Therefore, a lysozyme hydrophobic interlayer constructed on zinc metal can be used as a protective layer to suppress water-induced side reactions. Through electrostatic interaction, positive charges at the lysozyme layer and negative charges at the Zn anode significantly enhance the adhesion energy between them up to around 2600 J m^−2^ in a standard 180° peeling test (Fig. 5d). [37]

#### Liquid–Solid Transition

**Fig. 6:**
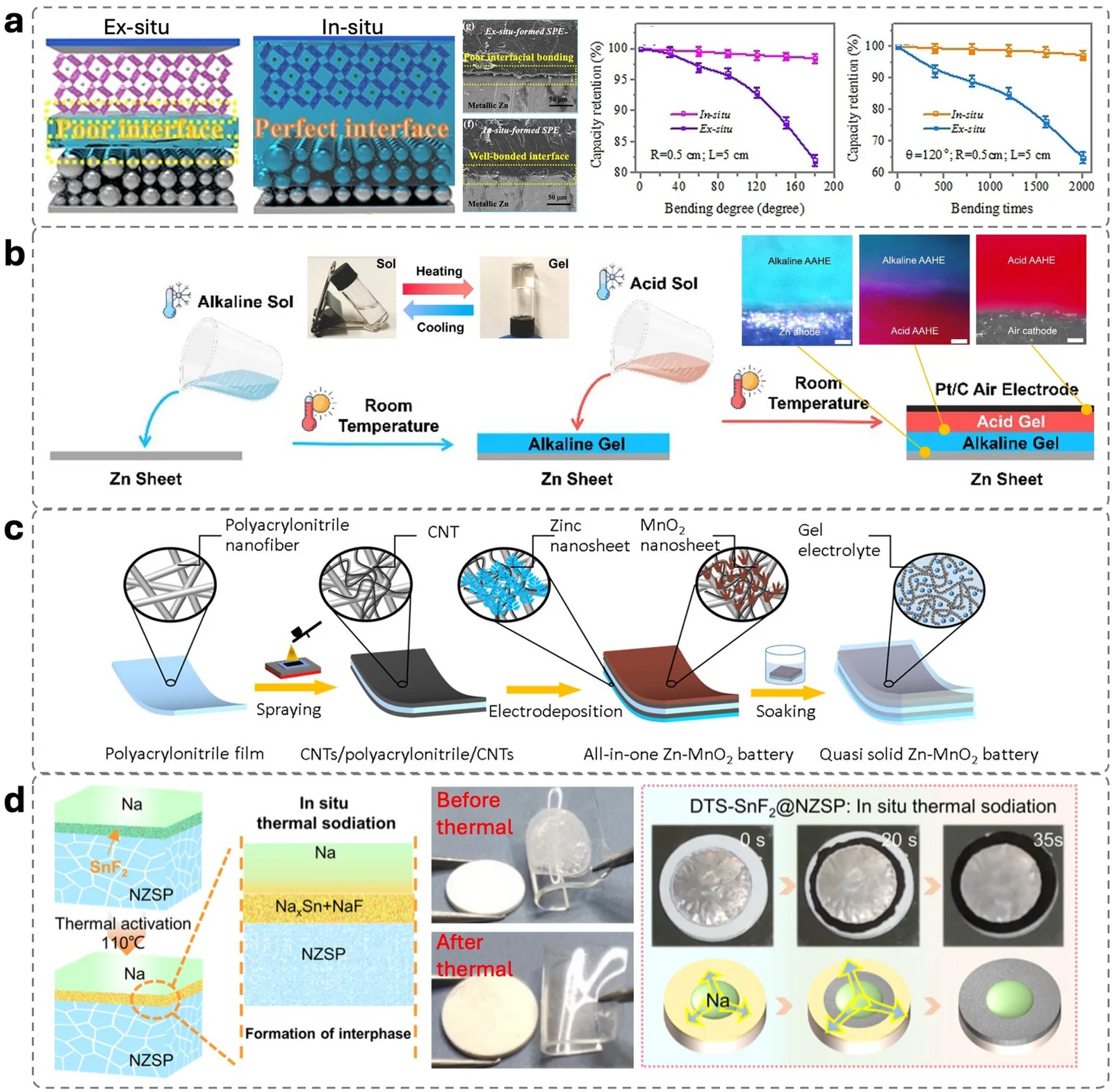
In situ liquid*–*solid transition for voidness interface.** a** Interface formation from ex situ and in situ solidification and the capacity retention versus bending degree and times [41]. Reproduced with permission. Copyright 2020 Wiley‐VCH GmbH. **b** Temperature-dependent liquid*–*solid transition [45]. Reproduced with permission. Copyright 2021 Elsevier B.V. **c** Separator-assisted all-in-one Zn-ion battery [47]. Reproduced with permission. Copyright 2021, American Chemical Society. **d** Utilizing in situ thermal solidification in an interlayer design [49]. Reproduced with permission. Copyright 2025 Royal Society of Chemistry

Liquid–solid transition, using flowable electrolyte precursor to fill the 3D structure or rough electrode surface followed by in situ polymerization or solidification, has been widely applied to maximize the contact surface area in conventional EES, and now extended to FEES (Model 1 and 3) for reinforcing dynamic contact. A solid-state electrolyte was in situ photopolymerized on a metallic Na electrode, which has similar impedance in flat and folding states, indicating good interfacial integrity and stability against deformation [38, 39]. Monomer precursor solution was cast on a separator and heat polymerized, immobilizing polysulfides owing to the high strength of the gel matrix and inducing a continuous and flexible passivation layer that buffers the volume change of sulfur particles during charge–discharge cycles [40]. In addition to chemical stability, the electrode–gel electrolyte interface has high physical adhesion; thus, a Li–S battery can power an LED under deformation, whereas a soft-packed battery with an electrode–liquid electrolyte interface has internal disconnection [40]. Satisfactory stability after bending cycles has been demonstrated in Zn-metal batteries with solid poly(1,3-dioxolane) and concentrated salt electrolyte. The advancement of the in situ polymerized interface against the physical contact interface (formed by sandwiching ex situ polymerized electrolytes between electrodes) was unveiled (Fig. 6a) [41]. By comparing capacity retentions and interfacial resistances at different bending angles and times, the superior stable contact under continuous deformation was found to be in situ polymerization, owing to the bonds and mechanical interlocks in the electrode–electrolyte interface formed during the polymerization process [41]. Unlike the ink-paste and metallic foils in Li-based batteries, textile-form electrodes based on carbon cloth substrate are popular in flexible Zn-based batteries. The liquid electrolyte precursor can penetrate the woven structure of such textiles and is then solidified by gelation. This process forms an all-in-one or integrated configuration, which has a simpler structure and more intimate contact than conventional layer-by-layer configurations [42, 43]. For example, the Zn- polyaniline battery, with an in situ polymerized polyacrylamide electrolyte, has achieved high-capacity retention after 3000 bending cycles [43].

Some polymer solutions alter their phase with temperature, namely the sol–gel transition. Aggressive bending and folding can break interfaces and disconnect the electrode and electrolyte; thus, recovery strategies have been developed that take advantage of temperature-dependent phase changes [44]. However, temperature dependence is a double-edged sword, because it can also restrict the applicable season and region. For portable devices, a wide range of working temperatures is necessary to meet the requirements of traveling. In addition, the distance between electrodes could change, potentially altering the battery performance and even leading to a short circuit. Thus, the electrochemical performance of these batteries at different temperatures with different (gel and liquid) states of electrolyte should be characterized. In addition to interface recovery, the sol–gel transition can simplify the fabrication of multilayer batteries. An all-in-one acid-alkaline Zn-air battery used a double in situ sol–gel transition (Fig. 6b) to form an alkaline Pluronic® F127 gel electrolyte on the anode, followed by an acidic gel electrolyte tightly attached by the air cathode, resulting in seamless contacts at the three interfaces and none of the distinct voids associated with a conventional layer-by-layer battery [45]. Further, the liquid–solid transition method can be extended to electrode modification [46]. For instance, Zn anode coupling with sol–gel formed Pluronic® F127 was demonstrated to obtain a large specific surface area and close interface contact with the gel electrolyte, leading to reduced passivation layer and interfacial resistance [46]. Sol–gel transition was also used in the separator-assisted gelation process (Fig. 6c), where the electrodes were electrodeposited on the two sides of the porous separator, followed by absorbing the liquid polymer electrolyte and in situ gelation [47, 48]. However, the electrolyte uptake of such a separator requires carefully control for results reproducibility and multiple interfaces could induce large polarization and a long ion transport pathway.

The key benefit of the liquid–solid transition strategy is that the intimate contact between the precursor solution and the electrode can be maintained by in situ transit into a solid or quasi-solid form. Achieving a void-less liquid–solid interface requires carefully optimizing the viscosity of the precursor solution and the surface affinity of the electrode toward the solvent. This strategy can also simultaneously be employed with the interlayer design as mentioned above, or in situ crosslink the polymer composition in a composite electrolyte [50, 51]. For example, a recent study utilized in situ thermal solidification to form a self-adhesive interlayer (Fig. 6d), where the wet spinning-coating enables a uniform contact area as the premise of the formation of adhesive joints in the followed thermal solidification. When the interlayer was thermal solidified with a Na anode, it presents good sodiophilic property, allowing uniform solidification and chemical reaction in different directions [49] The liquid–solid transition is commonly used for fabricating fiber-type FEES (see Reinforcement strategies for fiber-type FEES) combined with a dip-coating process [52, 53], because the fiber or spring shape of the electrodes is challenging to cover with pre-formed non-liquid electrolytes.

#### Adhesive Bonds

Establishing strong adhesive bonds or increasing the bond density is a straightforward method to improve the interface affinity between the hydrogel and the electrode and applies to all Models 1–4. Commonly used adhesive bonds/interactions are summarized in Table 1.

**Table 1:** Summary of common adhesive bonds

Bond/interaction	Examples	Key features
Covalent bond	Amide bonds, Ester bonds	Very high strength, permanent, forms chemical anchors
Hydrogen bond	–OH···O, –NH···O, –OH···N	Directional, reversible (moderate strength), water-sensitive
Ionic bond	–COO⁻···Na⁺, -SO₃⁻···Zn^2^⁺	Strong in dry state, salt/pH-dependent, reversible in water, directional
Dipole–Dipole	C = O···C = O, nitrile::nitrile	Moderate strength, directional, helps orient molecules
Electrostatic attraction	NH₃^+^⋯O⁻	Long-range attraction, tunable via pH/salt/charge density
Van der Waals	All neutral surfaces	Universal weak attraction, temperature-dependent
Metal–Ligand	Catechol···Fe^3^⁺, imidazole···Zn^2^⁺	Strong, dynamic, especially effective in wet environments, crosslinking
Hydrophobic	Alkyl–alkyl clustering in water	Nonpolar molecules aggregates to driven by water exclusion (entropy), important for wet adhesion
π–π Stacking	Benzene::benzene, pyrene::pyrene	Directional stacking of aromatic rings, moderate strength
Cation-π	Benzene:: NH₄⁺, Toluene::NMe₄⁺	Relatively strong, directional
CH-π	Alkyl C–H···Benzene	Weaker but common
Halogen bond	C–Br···O = C, C-I···N	Directional (C-X···D), moderate strength, tunable by halogen

**Fig. 7:**
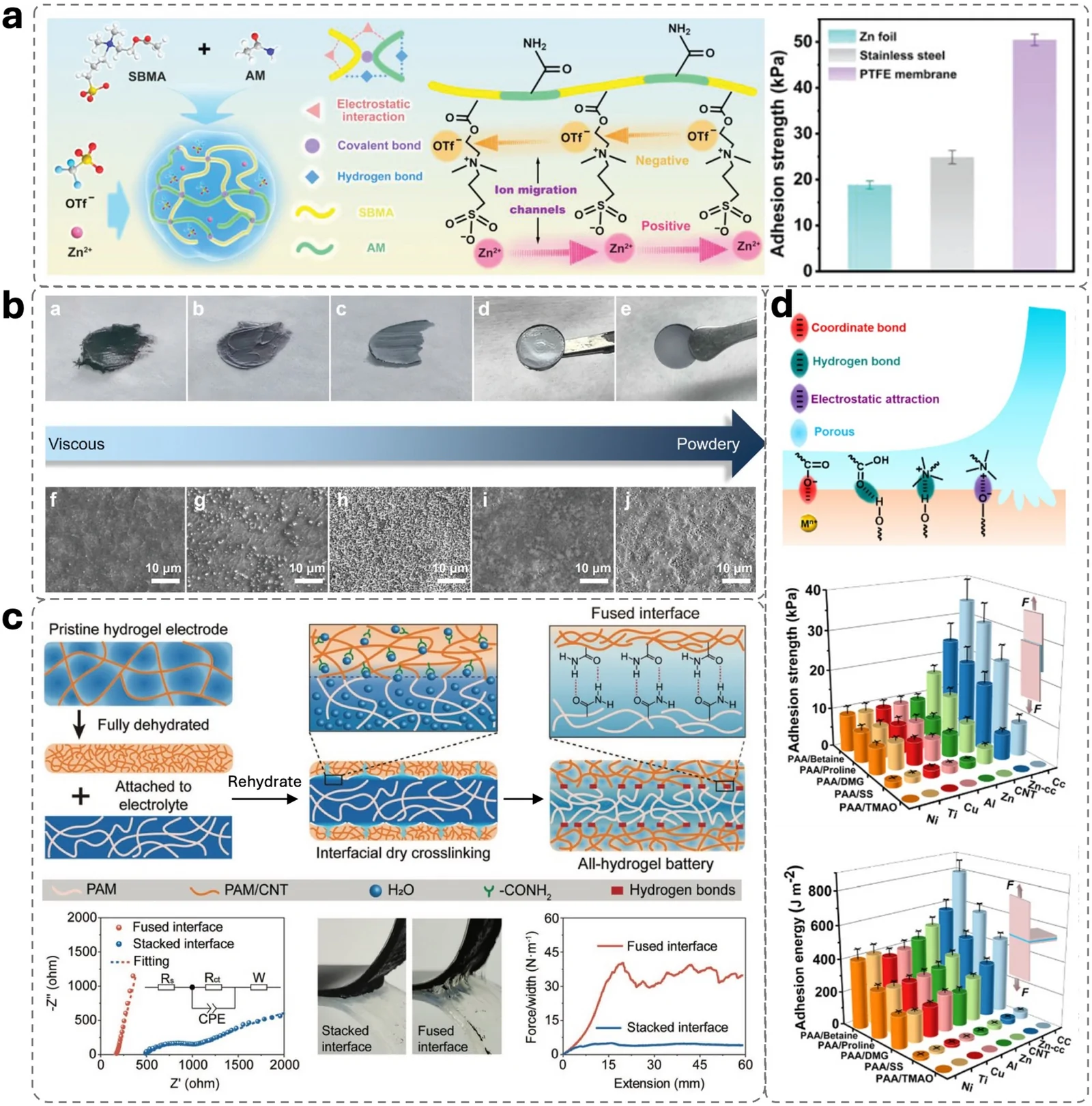
Adhesive bonds utilized in interface adhesion.** a** Multiple interactions induced by the copolymer network [58]. Reproduced with permission. Copyright 2022 Wiley‐VCH GmbH. **b** Halide-eutectic electrolytes with tunable viscosity and mechanical properties [62]. Reproduced with permission. Copyright 2022 The Authors. Advanced Science published by Wiley‐VCH GmbH. **c** Fused interface with high adhesion formed due to the densified interfacial adhesive bonds [61]. Reproduced with permission. Copyright 2022 Wiley‐VCH GmbH. **d** Multiple interactions induced by supramolecular hydrogel and its adhesion to various electrode materials [63]. Reproduced with permission. Copyright 2023 Royal Society of Chemistry

Covalent interaction has a high bond energy of 60–700 kJ mol^−1^ and widely exists in the crosslink of tough hydrogels, which have been employed on the interface between chitosan flexible sensors and skin [54]. In contrast, a reversible hydrogen bond with up to 40 kJ mol^−1^ bond energy has been proposed to support the intimate contact between hydrogel electrolyte and carbon- or hydrogel-based electrodes in flexible supercapacitors and metal-ion batteries [55, 56]. In addition, hydroxyl groups can constrain H_2_O contributing to interface stability at low temperatures. Because the high strain strength of hydrogels is a prerequisite for strong adhesion, dual-network [55] or elastic polymer [57] has been used to ensure the toughness of the whole flexible supercapacitor. Copolymers can incorporate various interactions, such as covalent and hydrogen bonds and electrostatic interactions, to enhance the mechanical strength and adhesion of electrolytes. The adhesion of a copolymer was evaluated with various adhered (Zn foil, stainless steel, and PTFE membrane) via the lap-shear test, which is in the range of 20–50 kPa (Fig. 7a) [58]. Recently, inspired by spider silk, malonic acid dihydrazide has been introduced to polyethylene glycol, leading to dense hydrogen bond formation owing to abundant -NH and = O [59]. Moreover, extending the hydrogel framework from electrolyte to electrode allows the stretchability and ionic conductivity of the electrode and offers an approximate elastic modulus and high interfacial hydrogen bonding density of the electrode and electrolyte [60, 61]. Deep eutectic electrolytes composed of molecules, ions, and/or molten salts can be synthesized without solvents, resulting in dense and complex hydrogen bonds, Lewis acid-based, and Van der Waals interactions. Therefore, their adhesion and cohesion properties can be tuned by adjusting the ratio of components to alter the strength of interactions (Fig. 7b). A viscous and dense halide-eutectic electrolyte with tunable mechanical properties can be used as a high ionic conductivity solid electrolyte, pasted as an interlayer to improve interfacial contact, or incorporated into electrodes as an ion-conducting additive, showing versatility for FEES application [62].

**Fig. 8:**
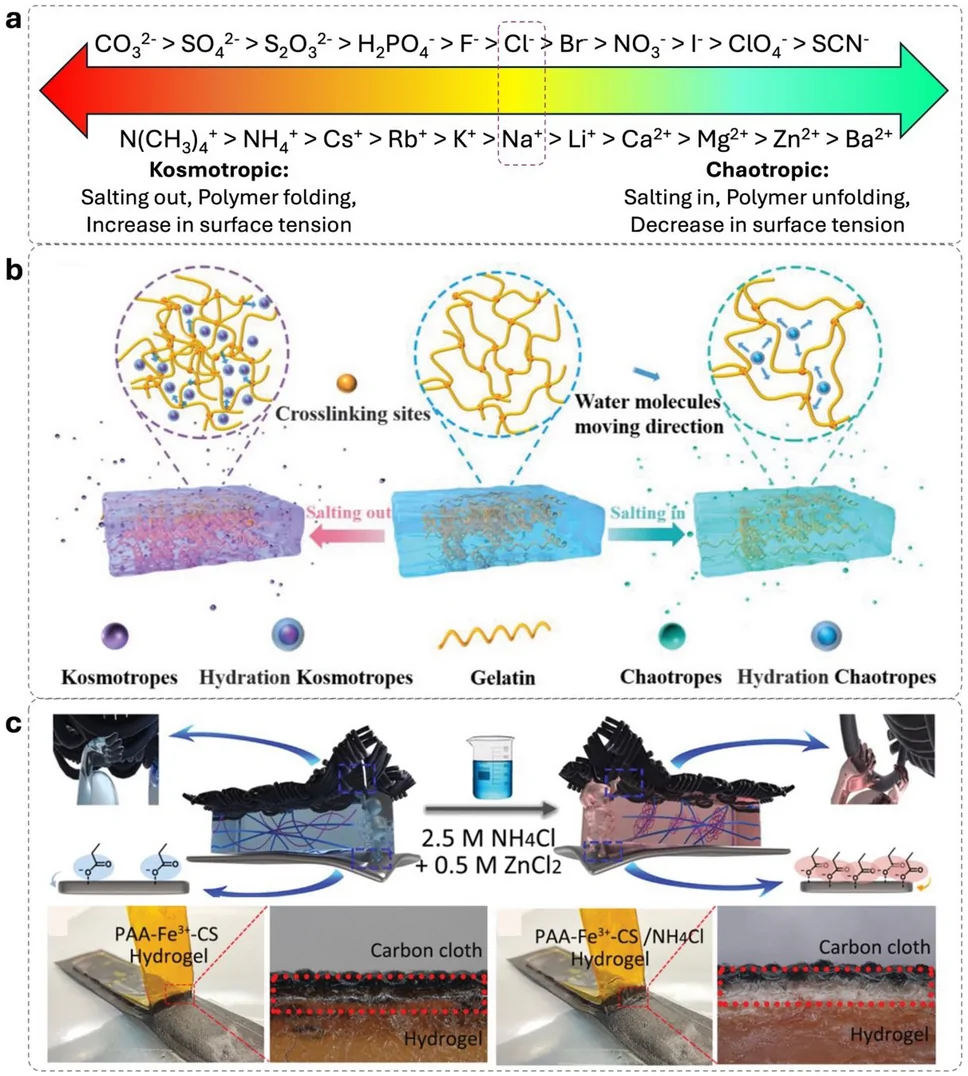
Hofmeister effect and its application in interface adhesion.** a** Hofmeister series. **b** “Salting in” and “salting out” in a polymer electrolyte [80]. Reproduced with permission. Copyright 2023 Wiley‐VCH GmbH. **c** Strengthening interface adhesion by post-soaking treatment in a kosmotropic NH^4+^ solution [70]. Reproduced with permission. Copyright 2021 Wiley‐VCH GmbH

The softness and high-water content of hydrogels result in a large contact interface with fast mass transport. However, surface water or wet conditions deteriorate the adhesion, leading to displacement or delamination. This loss of adhesion can be mitigated by water-in-salt, ionic liquid, and polymer densification approaches [39, 56, 61]. For example, a pre-dehydrated hydrogel electrode (the water was removed to increase the density of polymer, conductive agents, and adhesive joints) attached to a hydrogel electrolyte forms a fused interface due to the equilibrium of the aqueous solution in the two components (Fig. 7c) [61]. Hydrogels possess a structure of hydrophobic polymer chains decorated with abundant hydrophilic functional groups. Therefore, ions can alter the hydration shell of the functional groups changing the interface and mechanical properties of hydrogels. Hofmeister series is also valid in describing the ions tuning effect on polymer (Fig. 8a). Kosmotropic ions (strongly hydrated, such as CO_3_^2−^ and SO_4_^2−^) facilitate the formation of hydrogen bonds between polymer chains and strengthen the fold structure of the polymers increasing the density of crosslinking, functional group and polymer entanglement, while chaotropic ions (weakly hydrated, such as I^−^ and ClO_4_^−^) facilitate the break of hydrogen bonds and unfolding of the polymers leading to reverse effect (Fig. 8b). Thus, the Hofmeister effect has been used to manipulate the hydrophilicity/hydrophobicity [64–66], mechanical strength [8, 67, 68], and interface adhesion [69, 70] of hydrogels. A dual-network hydrogel consisting of polyacrylic acid and chitosan was soaked in a mixed solution of NH_4_Cl and ZnCl_2_ as a treatment to enhance the interface adhesion. The well-hydrated ions (NH_4_^+^ and Cl^−^) induced the folding of chitosan chains – that is, “salting out”—significantly increasing the density of carboxylate groups to act as chemical anchoring and thus boosting the adhesion between the electrolyte and electrode (Fig. 8c) [70]. A standard 90° peeling test verified the adhesion between the hydrogel (after soaking treatment) and a carbon cloth electrode, showing an adhesion energy of approximately 1001 J m^−2^, which is 9 times higher than that of the hydrogel without the soaking treatment. The adhesive interface decreases the charge transfer resistance (R_ct_) from 137 to 29 Ω and improves the mechanical durability of the device, which shows stable R_ct_ over 360 bending cycles, and the loose contact interface shows a significant R_ct_ increase after 60 bending cycles [70] Additionally, the salting out intensified the polymer entanglement, leading to volume shrinkage for which the ionic conductivity may be improved owing to the density increase of charge carriers. In general, ion transport is much faster in amorphous regions than in crystalline regions [71–73]. The strong interaction of kosmotropic ions and water rearranges the hydration structure in a higher order manner [65], leading to polymer aggregation and the increase of crystallization regions. Thus, the ionic conductivity is compromised with a further increase of the kosmotropic ions [69]. Meanwhile, salting out increases the bonding density, which affects the mechanical property of the hydrogel electrolytes, leading to high stiffness [8].

Ionic bonds and dipole–dipole interaction are used to strengthen adhesion, such as Zn^2+^ and S–O^−^ ionic bonds and Fe^3+^ ionic crosslinking [42, 74]. Both ionic bonds and dipole–dipole interactions exist on the interface of a hydrogel-based sensor and different substrates, exhibiting satisfactory adhesion [75], which is promising for FEES. Moreover, catechol chemistry can introduce multiple adhesive interactions [76, 77]. Dopamine-containing catechol groups are used to improve the shear adhesion strength between the electrolyte and electrodes [76]. Multiple interactions are introduced by a supramolecular zwitterionic hydrogel exhibiting strong adhesion to various electrode materials (Fig. 7d) [63]. It can be concluded that the adhesion between hydrogels and carbon-based electrodes is higher than that between hydrogels and metal electrodes owing to the mechanical interlock between the rough surface of carbon-based electrodes and the polymer chains of hydrogels [63]. Other than adhesion, the induced multiple functional groups also can constrain free water to regulate zinc plating/stripping chemistry [78], dynamically breaking and reconstructing bonds for self-healing and mechanical robustness, and wide-temperature adaptivity. This work also demonstrates the effectiveness of multiple interactions, provides comprehensive quantitative characterizations for future reference, and achieves significant success in the engineering aspect [63]. Similar concepts involving H-bonds, π–π stacking, cation–π interactions, and metal complexation also enhance the interfacial adhesion in an adenosine blended hydrogel, which is not only adhesive to electrodes but also self-adhesive [79]. A zinc-air battery with acidic-alkaline electrolytes has demonstrated a high open-circuit voltage of 2.15 V [79]. However, owing to the complex composition of these developed gel electrolytes, the adhesion mechanism study requires further exploration and advanced interfacial characterization.

Recently, standard adhesion tests were employed to qualitatively characterize the interface adhesion in Table 2 for ease of comparison and future benchmarking. Although other reviews summarized FEES performance including bending cycle, capacity, and capacity retention, to the best of our knowledge, this is the first and most timely review presenting recent progress in FEES from the point of adhesion science.

**Table 2:** Recent progress in interface adhesion for state-of-art FEES

Cell type (structure)	Electrolyte	Tensile stress (kPa)	Elongation at break (%)	Electrode	Shear adhesion strength ^1^ (kPa)	Adhesion energy ^2^ (J m^−2^)	Adhesion mechanism	Capacity and cycling assessment at flat state	Deformability assessment	Refs
Li-ion battery (sandwich, solid-state)	High-entropy PEO-based tape electrolyte with LiTFSI deep eutectic solution	8000	186	**Li ribbon**	**/**	**39.4**	Van der Waals, electrostatic attraction	~ 160 mAh g^−1^@ 0.1 C and 50 ℃ Cycling @ 0.1 C and 50 ℃ (20 cycles)	LED lighting at bending, twisting and folding	[81]
				*LiFePO*_*4*_* on Al foil*	*/*	*52.7*				
Li-ion battery (sandwich)	Poly(urethane-urea) polymer with Li_6.5_La_3_Zr_1.5_Ta_0.5_O_12_ and Li(TFSI)	~ 1750	900	**Graphite**	** ~ 100 **^**3**^	** ~ 390**	Hydrogen bonds	67 mAh g^−1^ @ 1C (20 cycles)	95.5% retention @ 1C (50,000 bending cycles)	[59]
				*LiCoO*_*2*_* on Al foil*	~ *120*	~ *420*				
Zinc-ion supercapacitor (coplanar)	Poly(AM-co-SBMA) hydrogel with 1 M Zn(TOf)_2_	27.9	315.4	**Zn on nylon membrane**	**18.8**	**/**	Covalent bonds, hydrogen bonds, electrostatic attraction	0.11 mAh cm^−2^ @ 0.6 mA cm^−2^ 94.6% retention @ 2.5 mA cm^−2^ (17,000 cycles)	Powering electronic watch by two-in-series devices	[58]
				*AC on nylon membrane*	*/*	*/*				
Zinc-ion capacitor (sandwich)	CNF crosslinked PAA with 2M ZnSO_4_ and EG additive	70.3	2150	**Zn**	**18**	** ~ 410**	Coordinate bonds, hydrogen bonds, electrostatic attraction, mechanical interlock	~ 140 mAh g^−1^@ 2 A g^−1^ 96.5% retention @ 2 A g^−1^ (50,000 cycles)	Various deformation at -20 and 60 ℃, respectively	[63]
				*AC on carbon cloth*	*34.3*	~ *800*				
Zinc-air battery (sandwich)	CMC crosslinked PAM-co-PANa hydrogel with 6M KOH and 0.2M ZnC_4_H_6_O_4_	292	2556	**Zn**	**46**	**/**	Coordinate bonds, hydrogen bonds	721.37 mAh g^−1^ @ 2 mA cm^−2^ Cycling @ 2.5 mA cm^−2^ (88 h) ^4^	No obvious degradation (60 cycles at 0°, 30°, 60°and 90° bending angle, respectively)	[82]
				*Carbon cloth*	*33.4*	*/*				
Zn-ion battery (sandwich)	PAM-xanthan gum hydrogel with ZnSO_4_ and glycerol	21	600	**Zn**	**1**	**/**	Hydrogen bonds	~ 140 mAh g^−1^ ~ 60% retention (100 cycles)	No obvious degradation (10 cycles at bent, compress and cut, respectively)	[83]
				*V*_*2*_*O*_*5*_* on stainless steel*	*2*	*/*				
Zn-ion battery (sandwich)	Dopamine grafted sodium alginate and PAAm-O double network hydrogel with 2 M ZnSO_4_ and 0.1 M MnSO_4_	383.2	1481	**Zn**	** ~ 74**	**/**	Hydrogen bonds, electrostatic attraction, π-π interaction	~ 150 mAh g^−1^ @ 4 A g^−1^ 91% retention @ 4 A g^−1^ (4000 cycles)	86% retention @ 0.5 A g^−1^ (500 cycles under bending state with a radius of 12 mm)	[76]
				*MnO*_*2*_* on carbon cloth*	~ *34.5*	*/*				
Zn-ion battery (sandwich)	Lysozyme inter layer on Zn with 2M ZnSO_4_ aqueous electrolyte	/	/	**Zn**	**/**	** ~ 2600**	Electrostatic attraction	125.7 mAh g^−1^ @ 10 C Cycling @ 10 C (2000 cycles)	No obvious degradation (5 cycles at 45°, 90°, 135°, 180° and 0° bending angle, respectively)	[37]
				*MnO*_*2*_* on carbon paper*	*/*	*/*				
Zn-ion battery (sandwich)	PAM-Hbimcp with 2 M Zn(CF_3_SO_3_)_2_	185.1	2650	**Zn**	**19.1**	**/**	Hydrogen bonds	230.6 mAh g^−1^@ 2A g^−1^ 75.2% retention @ 2A g^−1^ (1000 cycles)	163.4 mAh g^−1^@ 2A g^−1^ and no obvious degradation (450 cycles at 180° bending angle)	[84]
				*V*_*2*_*O*_*5*_* on carbon paper*	*/*	*/*				

Values in bold rows describe adhesion properties between adhesives and anodes. Values in ilatic rows describe adhesion properties between adhesives and cathodes

^1^ Shear adhesion strength is quantified by standard lap-shear test. ^2^ Adhesion energy is quantified by standard 180° peeling test. ^3^ Numbers with ‘ ~ ’ in front represent estimated values derived from graphs ^4^ Specific capacity of Zn-air batteries is calculated based on the mass consumed during the discharge process of the zinc electrode, while specific capacity of other devices is calculated based on the mass of cathode active materials

AM, acrylamide; AC, activated carbon; Ana, sodium acrylate; CMC, carboxymethyl chitosan; EG, ethylene glycol; Hbimcp, 2,6-bis((E)-(allylimino)methyl) − 4-chlorophenol; PAM, polyacrylamide; PAAm-O, poly(acrylamide-octadecyl methacrylate); PAA, polyacrylic acid; PEO, polyethylene oxide; SBMA, [2-(methacryloyloxy)ethyl]dimethyl-(3-sulfopropyl)

In summary, the five major adhesion reinforcement strategies exhibit distinct advantages and limitations depending on battery type, application, and mechanical requirements, as shown in Table 3. First, pressing is a simple and scalable strategy to enhance adhesion through polymer entanglement, but it is less applicable for mechanically fragile materials such as hydrogels. It is best suited for planar FEES with solid-state or composite electrolytes where pressure can be uniformly applied. Second, composite electrolytes improve mechanical strength and ion transport compared to polymer electrolytes, where the solid–solid interface can lead to poor contact and wetting, hindering strong adhesion. Additionally, strict thickness control is required to achieve flexibility. Thus, this approach is ideal for solid-state Li/Na-based FEES, particularly in applications requiring high-energy density with moderate flexibility. Third, artificial interlayers introduce customized interfaces to balance adhesion and electrochemical compatibility, particularly benefiting aqueous zinc-ion batteries where dendrite formation on the anode and cathode dissolution/shuttling represents distinct challenges. Moreover, it can also be employed in composite electrolytes for solid-state FEES to improve interface contact and adhesion. Likewise, interlayer thickness control is important for interface regulation to achieve adhesion and certain functions without significantly affecting bulk properties. Fourth, the liquid–solid transition begins with a liquid–solid contact that can be tuned by adjusting the precursor and substrate properties, e.g., surface energy and viscosity, followed by optimal solidification kinetics, in which a void-free and strong adhesion interface can be achieved. This strategy is promising for the electrode interface with various functional groups (for bond formation) or certain roughness (for mechanical interlock). Further, this strategy is highly compatible with coplanar cathode and anode configuration and fiber-type batteries with parallel electrodes or coaxial (layer-by-layer) configuration (see section “Reinforcement strategies for fiber-type FEES”. Finally, bonding via chemical or supramolecular interactions provides the highest level of adaptability, forming strong yet customized interfaces. It is compatible with aqueous systems as water is a good solvent for accommodating various solutes toward diverse bonding and interactions. However, tuning the bulk entails a trade-off between adhesion and other properties, such as ionic conductivity and mechanical properties, and therefore should be carefully considered.

**Table 3:** Comparison of adhesion reinforcement strategies

Strategy	Mechanism	Pros&cons	Cost	Complexity	Applicability	Durability
Pressing	Physical compaction; polymer entanglement;mechanical interlock	Scalable; improves initial interface contact	Low	Low	FEES with solid-state/composite electrolytes	Low Adhesion weakens under repeated deformation
		Not suitable for soft and fragile materials; limited durability				
Composite electrolytes	Improving contact; microinterlock; microstructure	High mechanical modulus; functionable	High (fillers costly)	Medium	Solid-state Li/Na FEES with high-energy–density but moderate flexibility requirement	Moderate Cracks form under large strain or repeated deformation due to brittleness
		Poor wetting at high filler load; limited flexibility and stretchability				
Artificial Interlayer	Improving interface compatibility and wetting; bonding formation; polymer entanglement	Locally applied; highly customizable and compatible; synergy of multiple adhesion mechanisms	Medium	High	Metal-based anode for suppressing side reactions/compatible electrode–electrolyte interface; sulfur or iodide cathodes for suppressing shuttle	High Maintains adhesion under repeated deformation due to synergy of multiple adhesion mechanisms
		Add processing step; precise thickness control; physical and chemical properties must be carefully optimized				
Liquid–solid transition	Mechanical interlock; bonding formation; polymer entanglement	Void-free contact; ideal for porous/rough electrodes; synergy of multiple adhesion mechanism	Medium	High	Versatile for various batteries; particularly suitable for porous metal electrodes or textile-based electrodes	High Maintains adhesion under repeated deformation due to synergy of multiple adhesion mechanisms
		Require surface energy/tension matching; viscosity optimization; solidification conditions sensitive				
Adhesive bonds	Bonding formation	Formation of reversible/irreversible bonds and reversible bonds are essential for high deformation degree and some functions like self-healing, self-assembly; strong adhesion; works in wet systems	Low to medium	Medium	Versatile for various batteries; particularly for aqueous flexible Zn-based batteries	High Maintains adhesion under repeated large strain deformation due to synergy of reversible and irreversible bonds
		Dense bonding may stiffen gel; sensitive to chemical environment, like ions and pH; reversible bonds may be sensitive to stimulus like temperature, light, moisture				

### Reinforcement Strategies for Fiber-Type FEES

**Fig. 9:**
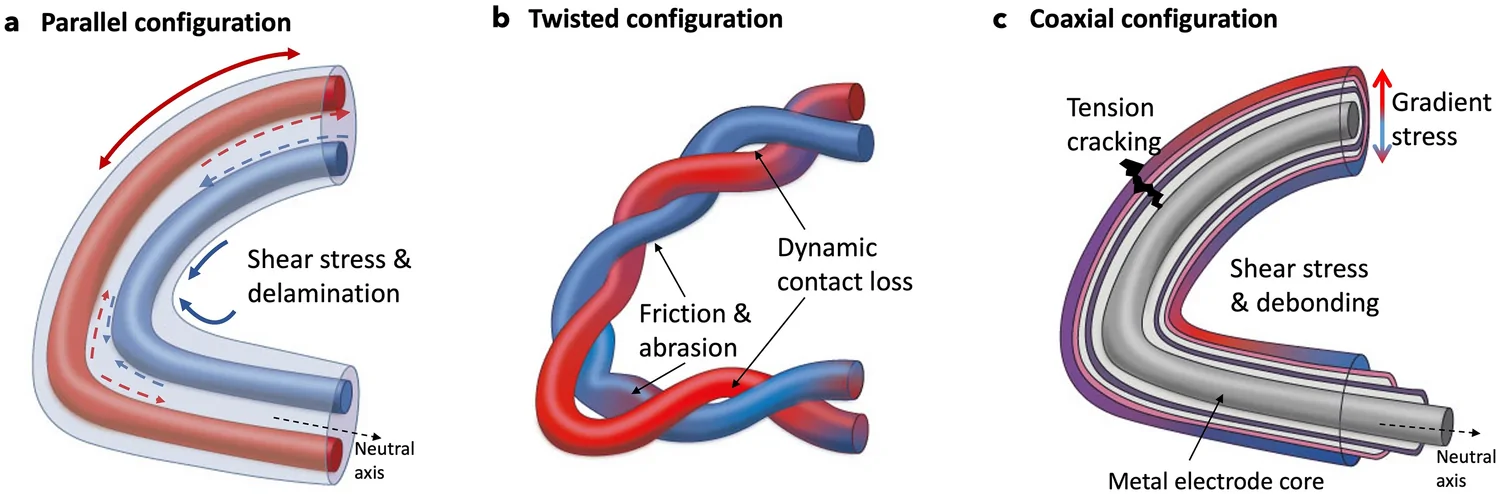
**a**–**c** Stress distribution and adhesion challenge of fiber-type batteries

Fiber-type FEES exhibits higher deformability than planar FEES due to its one-dimensional structure. Moreover, forming energy textiles with air permeability meets the wearing comfort. Stretchability can be realized by a topological or spring-like design. However, fiber-type FEES face unique challenges and the achieved capacity and cycling stability are still unsatisfactory compared to planar FEES. The urgent need lies in configuration design (e.g., for lower impedance, higher mechanical and electrochemical stability, and miniaturization) and fabrication simplification and optimization (e.g., for device uniformity, industrial-scale production, and quality control). The curvature interface of fiber-type FEES bearing stress along the surface even without bending poses strict requirements toward the mechanical strength of materials, and compatibility and compactness of the interface. Fiber-type FEES can be classified into three main configurations: parallel, twisted and coaxial, and their stress distribution and adhesion challenge of fiber-type batteries are depicted in Fig. 9. For parallel and twisted configurations, both liquid and gel electrolytes are applicable [84–86]. When using liquid electrolytes, a separator is required to avoid a short circuit. In contrast, gel electrolytes coated on electrode fibers simultaneously acts as a separator.

**Fig. 10:**
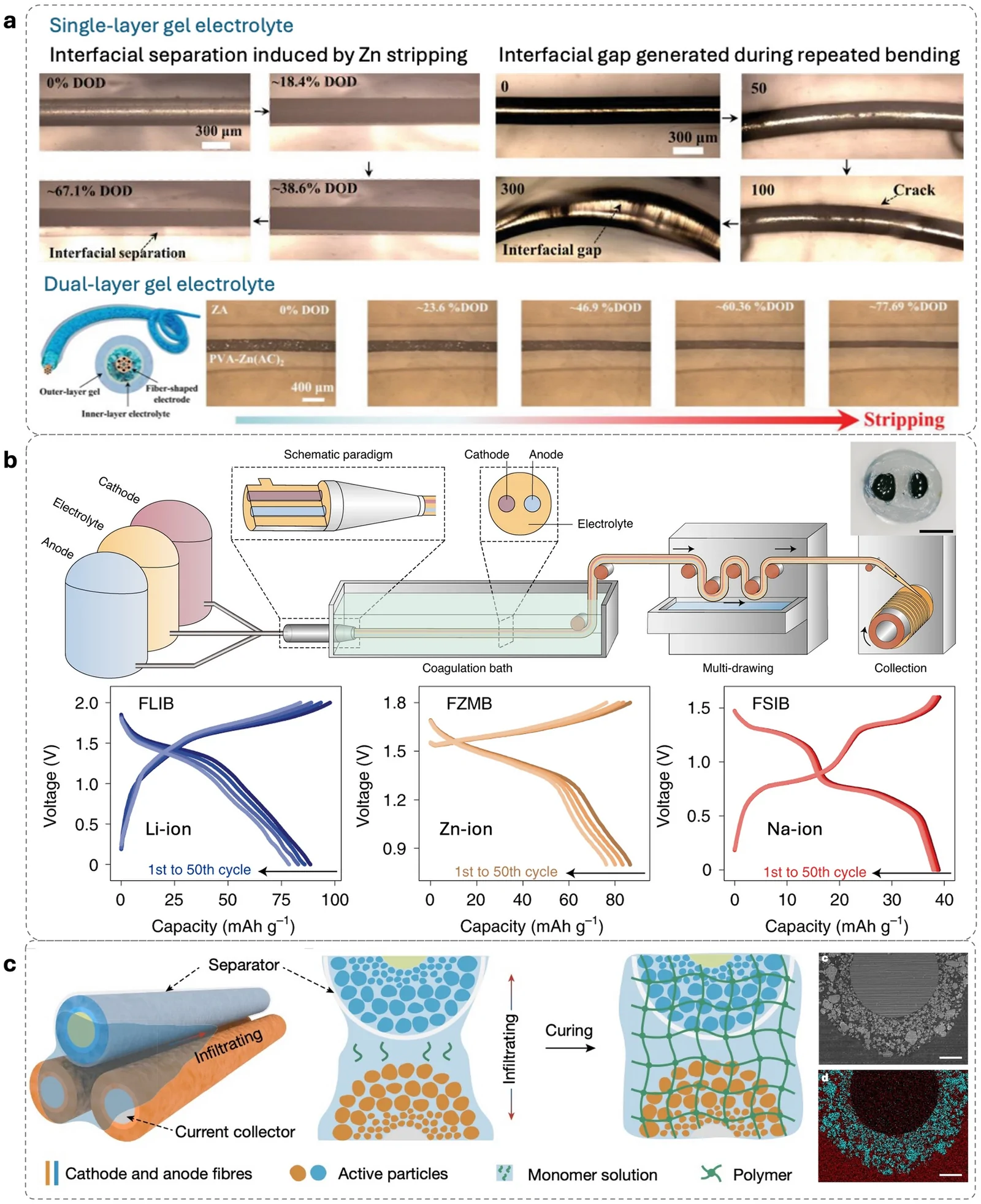
Interface challenges and reinforcements of fiber-type batteries.** a** Interfacial deterioration in a typical single-gel fiber-type FEES and dual-gel design [85]. Reproduced with permission. Copyright 2024 Wiley‐VCH GmbH. **b** Solution-extrusion method employing in situ polymerization for various fiber-type FEES [52]. Reproduced with permission. Copyright 2022 The Author(s) under exclusive license to Springer Nature Limited. **c** Gradient particles network for electrolyte infiltration and in situ polymerization [91]. Reproduced with permission. Copyright 2024 The Author(s) under exclusive license to Springer Nature Limited

For the parallel configuration, the stress distribution is anisotropic, heavily depending on the bending direction in relation to the electrode’s alignment direction. When the device is bent perpendicular to the line connecting the two fiber centers where the two fibers are bent in an equal manner, the compression is centered at the concave side, where volume shrinkage could crush the gel electrolyte or squeeze the liquid electrolyte leading to an ununiform distribution of electrolyte/ion. When the device is bent parallel to the connection line where the two fibers are bent in an unequal manner, one electrode experiences significant tensile strain while the other undergoes compression, which generates intense shear stress at the interface between the rigid electrode and the soft electrolyte. This mechanical disparity forces the surrounding gel electrolyte to accommodate a relative slip between the fibers. Consequently, the challenge is interfacial breakage and delamination or cohesive failure of the electrolyte, which provides insufficient ion transport and increases the risk of short circuit during harsh or repeated deformation conditions. Twisted fiber batteries employ a helical architecture that effectively converts macroscopic bending deformation into microscopic torsional strain, mimicking the mechanics of a spring to dissipate stress. While this geometry advantageously reduces peak tensile stress on active material compared to parallel or coaxial configurations, it introduces dynamic stress concentrations at the contact areas between the anode and cathode, where repeated bending induces micromotion and friction between twisted fibers. This dynamic micromotion leads to abrasion and detachment of active material and a fluctuating effective contact area that causes instability in the cell's internal resistance and capacity performance. Additionally, non-uniform coating thickness can form stiff regions that amplify stress concentration. Coaxial fiber batteries exhibit a radial gradient stress distribution similar to a classical composite beam, where mechanical strain scales linearly with the distance from the central neutral axis. As a result, the outermost electrodes are subjected to the highest stress, making them highly susceptible to tensile cracking on the convex side and/or compressive breakage on the concave side. The critical interfacial failure stems from modulus mismatch between the high-stiffness inner current collector, typically a metal wire, and the lower stiffness electrolyte, which drives detachment and interrupts the continuous ionic pathway essential for stable cycling. However, the interface adhesion in fiber-type FEES is rarely discussed due to the lack of effective, direct mechanical testing methods for cylindrical geometry. Conventional planar tests, such as lap-shear and 90° peel tests are not directly applicable to the interface of fibers. For this reason, most literature currently relies on electrochemical stability, such as resistance change under cycling, as an indirect indicator of interface quality. Looking forward, geometry-appropriate techniques, such as fiber pull-out, mandrel peel fixtures, and customized scratch tests, are necessary to quantify true adhesion energy and interfacial durability in fiber-type FEES. Establishing such rigorous mechanical protocols will enable a more critical comparison of adhesion strategies and actively promote the development of mechanically robust, next-generation fiber-based energy storage systems. A recent study demonstrates the interfacial deterioration in a typical gel-based fiber-type FEES induced by Zn stripping process and repeated bending (Fig. 10a) [85]. The stripping of Zn causes shrinkage of the Zn fiber anode leading to interfacial separation. Gel electrolytes generally possess high flexibility and elasticity owing to low elastic modulus in the range of 10^3^–10^7^ Pa [86, 87], while zinc has a high elastic modulus of approximately 108 GPa [88]. Due to the mismatch between the elastic modulus of Zn anode and common gel electrolytes, repeated bending causes interfacial gaps and cracks. Dual-gel design with a soft and creep interlayer between the Zn anode and the typical gel electrolyte was proposed (Fig. 10a), where the high mobility of the interlayer can rapidly fill the interfacial gap while the outer gel electrolyte with a relatively high strength can protect the cell from short circuit. This approach can be classified into the above-discussed artificial interlayer. In general, the integrity and interface contact are easily deteriorated by “point-defects” during repeated deformation because the bending force is centered on a point. Self-healing relying on reversible bonds can assist repairing interface and device damage [89, 90].

Liquid and gel electrolytes were compared in a parallel Zn-ion fiber battery, where the interfacial resistance of the battery employing liquid electrolyte more aggressively increased after 300 charge–discharge cycles than the gel indicating a more stable gel-electrode interface [92]. However, the tests were carried out without bending. Thus, the interface variation is not caused by deformation; instead, it may indicate that the electrochemical stability of the curvature interface requires mechanical support from the gel electrolyte. A scalable and continuous fabrication of twisted Li-ion fiber battery was demonstrated involving dip-coating, wrapping, twisting, and encapsulating [93]. The assembled battery delivered comparable electrochemical performance to typical Li-ion batteries as well as a mechanical strength comparable to textile fibers, enabling it to be woven by an industrial rapier loom [93]. Compared with parallel and twisted configurations, the coaxial configuration provides a larger effective surface area and tighter interface, which allows high utilization of active materials and structural stability to withstand frequent deformation. In the coaxial configuration, the adhesiveness of gel electrolytes determines the firm attachment between the anode and cathode. Stretchability can be achieved by using a spring Zn electrode followed by coating with an adhesive electrolyte and wrapping with cathode material [94]. A coaxial Zn-ion battery incorporating multilayers of carbon nanotube sheets and electrolytes delivered excellent performance against bending and 93.3% capacity retention after 3000 bending cycles [95]. Wrapping carbon nanotube sheets may not only favor fast charge transport but also provide a compression force to keep the layers in close contact, leading to decent performance in deformation tests. Although a process for continuous production has been proposed [95], the fabrication of coaxial fiber-type FEES is generally more tedious than the parallel and twisted configuration [96]. Electrode fibers can be wound onto a stretchable substrate forming a wound configuration with higher stretchability. However, the ability to withstand repeated stretching remains an issue and the capacity decay mechanism may be different from bending, requiring further study [97]. Post-winding can realize device-scale stretchability for fiber-type FEES in all configurations.

Dip-coating is a commonly used method in fabricating fiber-type FEES, which has the merits of high scalability and cost-effectiveness with abundant guidance from the textile industry. However, the relatively small active materials loading, poor uniformity, and inadequate interface stability remain challenging, since the curved surfaces of fibers lead to high surface tension for coating slurry [93]. Appropriate binders/surfactants should be explored to optimize the coating quality, including the affinity of the coating slurry and substrate, and the uniformity and durability of the coating. 3D printing was employed to fabricate cathode and anode yarn fibers showing potential for scalable production [98, 99]. A solution-extrusion method employing a three-channel industrial spinneret to integrate electrodes and electrolytes in a single step to form parallel fiber batteries with a diameter of 500–1000 µm was demonstrated, where the interface formation can be classified into the above-discussed in situ liquid–solid transition with the merits of intimate contact and durable connection (Fig. 10b) [52]. The electrodes and electrolyte are tailorable by adjusting the composition and viscosity of the precursor solution to adapt to different batteries, such as Li-ion, Na-ion, and Zn-ion batteries (Fig. 10b), and ensure the spinnability and compatible mechanical properties of components, resulting in higher interface stability. Similar wet spinning was employed to fabricate V_2_O_5_/rGO hybrid fiber cathode for Zn-ion battery, which suggested the rGO provides a wrapping protection to V_2_O_5_ nanowires resulting in higher interfacial adhesion stability that can withstand 500 bending cycles exhibiting significant contrast to the cathode fabricated by dip-coating. Meanwhile, the wet-spun fiber delivers an impressive specific capacity of 486 mAh g^−1^ comparable to planar zinc-ion batteries. Nevertheless, capacity degradation still occurs after 500 bending cycles. A fiber electrode with a gradient particle size forms interconnected channels (Fig. 10c), which benefit the infiltration of monomer solution, followed by in situ polymerization, leading to an intimate and stable electrode–electrolyte interface. The interconnected channels have high porosity that enlarges the contact surface and potential adhesive joints, and the surface roughness enhances the mechanical interlock with polymer chains. As a result, the stable electrode–electrolyte interface renders the fiber-type FFES > 96% capacity retention after 100,000 bending cycles [91]. There is no doubt that fiber-type FEES has higher structural flexibility than planar batteries; however, the mechanics and factors that influence electrochemical stability under deformation are more complex. The decay mechanism of fiber-type FEES induced by deformation requires extensive research efforts.

### Adhesion Mechanism

**Fig. 11:**
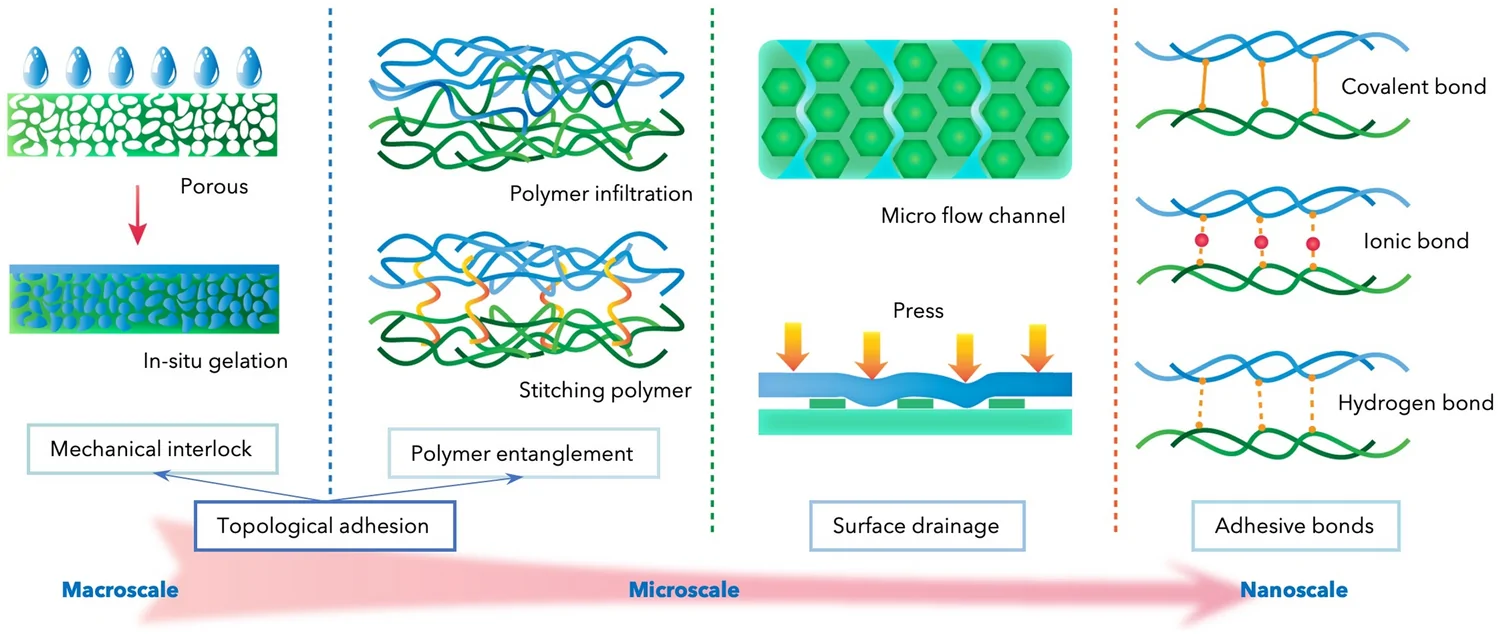
Schematic illustration of multiscale adhesion mechanism in FEES

Impressive progress in the adhesive interface has been achieved using quasi-solid electrolytes, including gel and hydrogel electrolytes for non-aqueous and aqueous systems, respectively. However, the mechanism interpretation is still in the infant stage, lacking a comprehensive review and insightful analysis. Herein, based on the current strategies in FEES and inspiring methods in other applications, such as biomedical applications [77, 100], soft electronics [101], and underwater taping [102, 103], the underlying adhesion mechanisms are discussed from macro to nanoscale (Fig. 11).

For the macroscale, electrode or electrolyte surface structure can contribute to the commonly employed mechanism in dry conditions, namely mechanical interlock. Although the deformable gel and hydrogel electrolytes can escape from the key-lock topology, the in situ gelation of electrolytes inside the porous structure of the electrode, such as carbon cloth and etched elastomer, will realize the mechanical interlock. Even on rough surfaces without pores, the increased interfacial area can still enhance the adhesion, facilitating the interface stability at small scale deformation by raising energy dissipation [21]. Moreover, the topological adhesion mechanism at the polymer scale has also been discovered and is applicable to wet conditions, which can be achieved by diverse combinations of bond and stitch topology [21, 104]. Pressing and decreasing water content can increase the polymer entanglement density. In addition to minimizing the bulky water content, surface drainage can eliminate the negative effect of hydration layers [105]. Such surface engineering at the microscale is bio-inspired by tree-frog and clingfish, promising for hydrogel adhesion [102, 106]. In addition to the interface architecture design, involving highly hygroscopic polymers in the network can also effectively absorb the surface water. A tape-like adhesive electrolyte was achieved due to the high entropy of polymer chains and water-free nature, resulting in interface adhesion energy of 39.4 and 52.7 J m^−2^ with Li ribbon anode and LiFePO_4_ cathode, respectively. Owing to the sufficient adhesiveness of the high-entropy polymer electrolyte, the battery assembly can be simplified by adhering electrodes to the electrolyte, without high-compression pressing, simultaneously generating an integrated interface with good contact [81]. For the nanoscale, the bonds between electrolyte and electrode dominate the adhesion mechanism. Although covalent bonds are common for underwater adhesion due to the higher bond energy compared to physical bonds, they have not been widely used for FEES. Other physical interactions, such as hydrogen and ionic bonding, have been successfully established to enhance the hydrogel–electrode interface adhesion in FEES.

Furthermore, the synergy of different adhesion mechanisms ranging from macro to nanoscale can ensure intense dynamic contact in FEES. A typical demonstration of multiscale adhesion synergy is the in situ gelation of polymer electrolytes within carbon cloth electrodes [16, 43]. At the macroscale, the polymer precursor penetrates the woven textile structure, forming mechanical interlocking. After solidification, the electrolyte forms conformal contact leading to an increase in surface roughness, which increases the interfacial contact area and provides additional adhesive joints. At the microscale, the polymer network that forms around the fiber establishes topological adhesion through chain entanglement. Pressing at the packaging stage causes volume shrinkage, which enhances the polymer entanglement density. At the nanoscale, polymer chains form adhesive bonds, such as hydrogen bonding and ionic coordination, with functional groups on the carbon fiber. Together, these multiscale adhesion mechanisms reinforce each other to maintain stable contact under deformation, and demonstrate that multiscale synergy can substantially enhance interfacial robustness in flexible devices. Such synergetic adhesion mechanisms have not been well interpreted in literature. Further research and application of the adhesion mechanism will speed up the practical deployment of FEES. In addition, characterizing the interface affinity with those enhancement approaches is straightforward by measuring the mechanical adhesion strength and toughness [21]. In contrast, assessing the impact of the reinforced dynamic contact on FEES performance is still under development. Therefore, the evaluation of FEES performance is discussed below.

### Evaluation of Flexibility and Durability

**Fig. 12:**
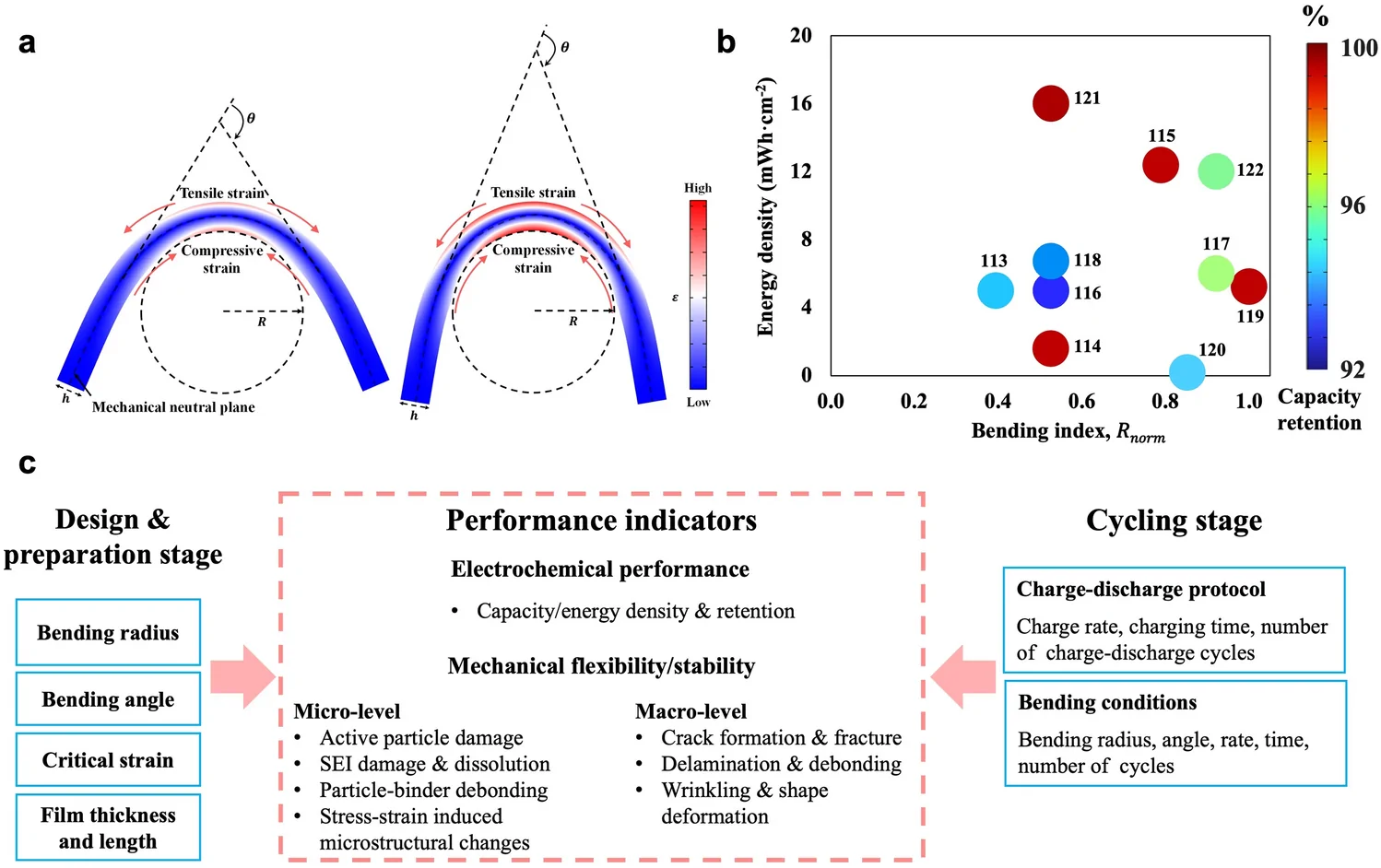
Bending parameters, bending index and multicriteria assessment framework. **a** Schematic representation of bending mechanics in a deformable battery. Deformation affects FEES’ electrochemical performance. Bending radius $\left(R\right)$ and bending angle $\left(\theta \right)$ influence the stress distribution and film with higher θ exhibits higher strain as compared to the lower θ film with similar R. Device geometry also influences the bending mechanics, where the local displacement with the same θ can be distinguished for the FEES with different thicknesses and lengths. **b** A comparison plot of various flexible batteries with respect to areal energy density, bending index, and capacity retention (capacity retention is relative capacity in percentage after > 1000 repetitive bending cycles) [112–121], and **c** multicriteria assessment framework including key performance indicators and deformation parameters for device design and evaluation

Deformation state and device geometry affect FEES’ electrochemical performance (Fig. 12a). Unfortunately, these variables are not comprehensively considered in prior studies. Thus, it is crucial to gain a consensus on assessing FEES performance under deformation. We believe that evaluating FEES based on standardized bending criteria driven by application is important to promote the practical adoption of FEES. To enable application‐driven and standardized assessment of FEES flexibility, we introduce a normalized bending index (${R}_{norm}$) that compares the device’s bending capability with the deformation window required for practical applications. For instance, wearable electronics typically require operating bending radii between 1–20 mm and durability beyond 1000 bending cycles (Table 4).

**Table 4:** Desired mechanical deformation parameters for wearables [107–110]

Parameter	Required range	Applications
Bending radius	1–20 mm	Medical patch, smart watch belt, wearable heater, smart gloves, healthcare, and athlete performance monitoring devices
Bending angle	30–180 degrees	
Bending cycles	> 1000	
Stretchability^*^	30%–100%	

However, most FEES studies report bending radii only under laboratory-selected conditions, without reference to the practical thresholds, making cross-study comparison difficult. To resolve this, we define the bending index as $R_{norm} = \frac{{R_{a,max} - R_{d, act} }}{{R_{a,max} - R_{a,min} }}$, where ${R}_{d, act}$ is the minimum bending radius a device can withstand, and ${R}_{a,\mathrm{min}}$ and ${R}_{a,\mathrm{max}}$ correspond to the minimum and maximum bending radii required for wearables. A value of **1** indicates the device meets the minimum bending requirement, whereas values > **1** indicate additional flexibility margin. This index therefore provides a clear, application-centered criterion for determining whether a device satisfies the mechanical requirement, which is valuable for quality control, product development, and comparative analysis among different devices for certain applications, i.e., wearable scenarios. Based on the bending radius in Table 4, the bending index, areal energy density, and capacity retention (tested for > 1,000 bending cycles) of state-of-art FEES are plotted in Fig. 12b. All data points in Fig. 12b are extracted from the cited references, using reported bending radii to compute the corresponding application-normalized bending indices, estimated areal energy densities, and > 1000-cycle durability results. It is also important to note that the bending index does not replace the Figure of Merit (FOM), which evaluates the efficiency of balancing energy density and flexibility [107]. Instead, the two metrics serve complementary roles, where FOM captures performance trade-offs while the bending index standardizes mechanical compliance with application-specific requirements. Additionally, the ability of a wearable device to stretch and deform in synchronization with the body is crucial for users’ comfort and device functionality. For example, on the skin, a wearable device needs to exhibit a stretchability of around 30% to comfortably conform to the body’s movements [109]. On joints, the device needs to possess even higher stretchability, exceeding 100%, to allow for a full range of motion without compromising device integrity [110].

The multiphysical phenomena, such as charge transport and stress distribution under deformations, influence the output of FEES, which is challenging to visualize in experiments, especially under dynamic conditions. The finite element method can interpret the interplay between electrochemical processes and mechanical deformation for structure design and analyzing potential failure modes [111].

Overall, the electrochemical and mechanical performance is influenced by multiple factors, leading to the lack of impartial comparison among diverse FEES. We propose a multicriteria assessment framework, combining the crucial mechanical parameters and performance indicators to evaluate FEES under deformation (Fig. 12c).

## Outlook

**Fig. 13:**
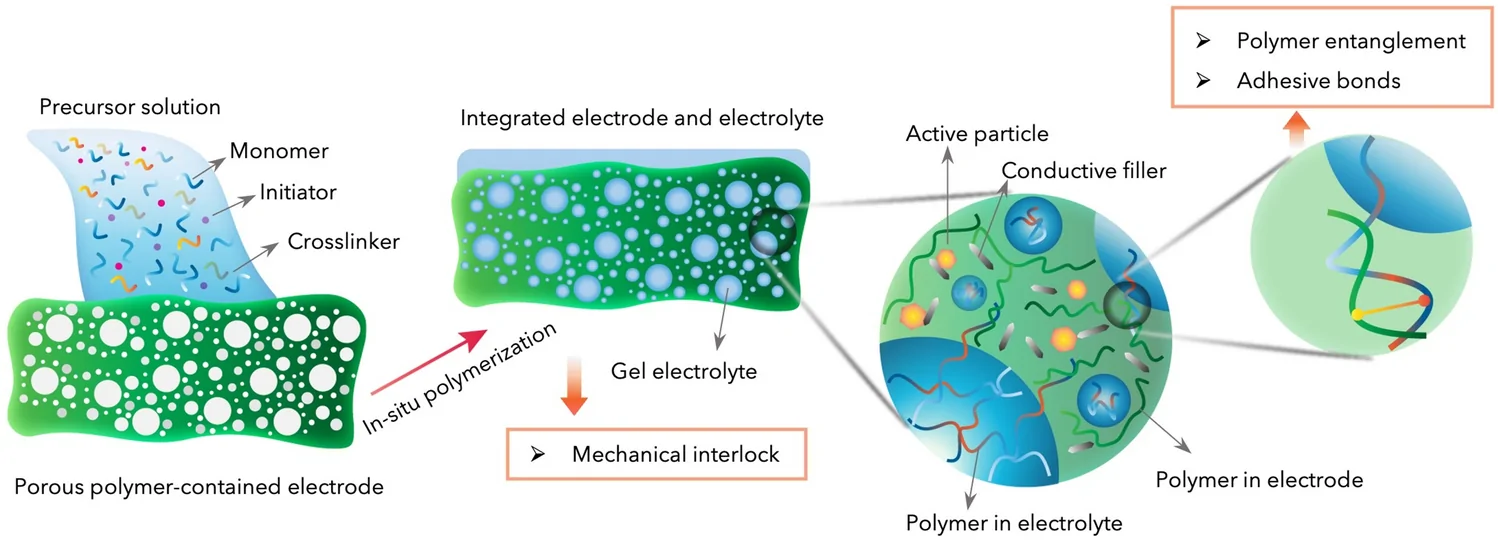
Perspective on future interface construction. In situ polymerization of gel electrolyte in porous polymer-contained electrode toward different adhesion mechanisms combination

Delamination-free electrode–electrolyte interface under deformation represents the basis for the practical application of FEES. Based on the above analysis, in situ polymerized gel or hydrogel electrolyte on elastic porous electrode is recommended for further research to achieve durable FEES (Fig. 13), which incorporates mechanical interlock, polymer entanglement, and bonds. For instance, a porous electrode can be built through freeze-drying a hydrogel-based electrode, which can maintain the porous structure and subsequently absorb the monomer solution, where the monomer permeates into the solid framework, then polymerizes and entangles with the polymeric electrode. Simultaneously, the adhesive bond can be devised by selecting the polymer type and/or dopant.

So far, the fundamentals of the adhesive electrolyte and their interrelation with FEES electrochemistry are deficient. We propose the following research directions to study the electrode–electrolyte interface in FEES and ameliorate the dynamic contact and adhesion under deformation.

- **Ion environment**. The knowledge transfer of adhesive hydrogels in biomedical and underwater applications could be affected by the ion environment of FEES. For instance, the energy carriers and counter ions in zinc-ion batteries influence the electrostatic interaction, such as hydrogen and ionic bonds, between hydrogel electrolytes and electrodes. Besides, organic and water co-solvents or water-in-salt have been used to eliminate water-induced side reactions or attain anti-freezing ability, but their influence on interface adhesion remains missing. The impact of salt type, concentration, and solvent on the hydrogel adhesion will inform the exploration of superior electrolytes for FEES.
- **Local environment**. Long-term charge-discharge cycles affect the interface physics and chemistry, consequently altering physical contact and adhesion. For example, voids form following dendrite stripping or bubble generation diminishing the physical contact. Hydrogen insertion changes the local pH of the cathode–electrolyte interface, affecting the adhesive bonds. Therefore, a quantitative assessment is required to predict and regulate these dynamic interface effects. Advanced characterizations, such as visualizing the dynamic interface variation via cryogenic electron microscopy, provide a good start in this direction. Chemically robust interlayers (pH-buffer adhesives) can be applied to resist local chemistry changes and suppress side reactions.
- **Ambient environment**. Polymers change their mechanical stability and adhesive strength under high humidity or elevated temperatures. This also applies to the adhesion and sealing function of the packaging layer. Environmental effect alters the layer adhesion, leading to delamination and loss of structural integrity. For the packaging layer, wide-temperature adaptability and low permeability of air and moisture protect the cell from environmental effects to some extent. Developing soft and wide-temperature adaptative adhesion is critical to ensure the cell maintains an intrinsic close contact under varied environments and deformation.
- **Interface construction.** Soft adhesives wet electrodes well, while highly crosslinked adhesives exhibit poor wetting and fail to penetrate micro and nanoscale structures. This creates an intrinsic conflict between achieving intimate initial contact and ensuring long-term mechanical robustness. In situ gelation offers a pathway to reconcile this trade-off, in which precursor liquids with low-surface tension can first infiltrate electrodes and subsequently undergo crosslinking to form a mechanically robust network, representing a future trend of FEES. However, the properties of precursor solutions, including surface tension and viscosity, are crucial for such fabrication processes and are rarely discussed in current research. Rheological characterization of the gelation process should be used to evaluate the impact of precursor solution and sol-gel transition on the interfacial contact. In addition, SEI is essential, particularly for Li/Na-based batteries. Repeated bending causes the electrode surface to experience tension and compression, breaking the fragile and brittle SEI layer. The exposed fresh electrode consumes electrolyte to continuously reform the SEI, leading to irreversible electrode and electrolyte loss, increased interfacial resistance, and capacity fade. Thus, developing an elastic and self-healing SEI that is compatible with FEES is critical to improve the durability of FEES.
- **Interface uniformity.** In FEES manufacturing, maintaining a uniform interface across large areas or long fiber lengths is inherently challenging. Non-uniformities in coating thickness, surface porosity, or drying rates create local variations in mechanical modulus and interfacial morphology. Even before considering adhesion, geometric and material heterogeneity lead to uneven strain distribution during deformation, promoting local cracking. When these morphological variations coincide with inconsistent interfacial adhesion, which is common in large scale processes due to fluctuations in surface cleanliness, wetting behavior, and processing local conditions, the resulting strain mismatch becomes amplified. Weakly bonded spots become preferential sites for peel-driven delamination, interfacial shear failure, and/or progressive void growth under cyclic deformation, ultimately causing mechanical and electrochemical instability. Thus, developing materials and adhesion strategies that tolerate or compensate for inevitable manufacturing heterogeneity is important. For example, interlayers that redistribute strain, or processing protocols that actively monitor and regulate coating uniformity. Addressing both geometric and adhesion uniformity is essential for translating FEES from small laboratory samples to reliable and scalable energy storage devices.
- **Interface examination.** The effectiveness and durability of the interface adhesion are closely linked with battery performance. Thus, electrochemical indicators were used to reflect the interface property. However, using indirect indicators makes the assessment of interface adhesion ambiguous since multiple influencing factors are involved. Interface adhesion in flexible batteries is an emerging interdisciplinary research direction, which involves adhesion science, deformation mechanics, and electrochemical surface science. To fairly compare adhesion strength (without the impact from the surface property of the adherend), AFM should be employed as a standard method to quantify the surface adhesion, followed by typical adhesion tests, such as tensile, lap-shear, and peeling tests. In addition to these tests, fatigue and environmental tolerance tests should be employed to study the interface adhesion in FEES, because durability and wide environmental adaptability of the adhesive determine the durability and wide application of FEES.
